# Effect of probiotics on necrotizing enterocolitis in preterm infants: a network meta-analysis of randomized controlled trials

**DOI:** 10.1186/s12887-025-05469-z

**Published:** 2025-03-27

**Authors:** Yu Dai, Qinlei Yu, Fan Zhang, Ke Ma, Xiangyun Yan, Wenjuan Chen, Xiaohui Chen, Shushu Li, Shuping Han

**Affiliations:** Department of Pediatrics, Women’s Hospital of Nanjing Medical University, Nanjing Women and Children’s Healthcare Hospital, 123 Tian Fei Xiang, Mo Chou Road, Nanjing, 210004 China

**Keywords:** Probiotics, Feeding intolerance, Necrotizing enterocolitis, Preterm infants, Network meta-analysis

## Abstract

**Background:**

Previous studies have suggested that probiotics may have potential benefits for preterm infants. Their efficacy seems to depend on the particular species or combinations used.

**Methods:**

To further investigate the effects of probiotics in preventing necrotizing enterocolitis (NEC) and other related outcomes in preterm infants, we conducted a network meta-analysis of 51 randomized controlled trials involving 11,661 participants.

**Results:**

Our study revealed that most probiotics can effectively reduce the incidence of NEC (at or beyond Bell’s stage II). Lactobacillus (RR, 0.59; 95% CI: 0.29, 0.98), the combination of Bifidobacterium and Lactobacillus (RR, 0.47; 95% CI: 0.20, 0.87), and the combination of Bifidobacterium, Lactobacillus, and Streptococcus (RR, 0.17; 95% CI: 0.00, 0.84) were the only treatments that significantly reduced all-cause mortality compared to placebo. Lactobacillus can be effective in reducing the time preterm infants spend in the hospital (MD, -4.23; 95% CI: -7.62, -0.81) and reaching full enteral feeding (MD, -2.15; 95% CI: -3.70, -0.64).

**Conclusions:**

The combination of Bifidobacterium, Lactobacillus, and Enterococcus was the most efficacious in reducing the mortality and incidence of NEC (Bell II or above) in preterm infants. Both prebiotics and Lactobacillus alone were found to be highly effective in reducing the length of hospitalization and the time needed to achieve full enteral feeding. No evidence suggests that probiotics affect sepsis risk.

**Trial registration:**

The study protocol was registered with PROSPERO (CRD42023460231) on March 10, 2023.

**Supplementary Information:**

The online version contains supplementary material available at 10.1186/s12887-025-05469-z.

## Background

With advancements in medical standards, a growing number of preterm infants are able to survive. However, the burden of disease associated with preterm birth remains severe and has become a crucial contributing factor to the mortality of children under five years old globally [1]. These infants are particularly susceptible to gastrointestinal disorders due to the immaturity of their organ systems, prolonged exposure to the hospital environment and the early initiation of antibiotic treatment [2]. As gestational age and birth weight decrease, the incidence of feeding intolerance (FI) increases [3]. FI hinders premature infants from obtaining sufficient nutrition, which can even lead to long-term growth restriction and neurodevelopmental disorders [4]. Severe FI may result in complications such as necrotizing enterocolitis (NEC) and neonatal late-onset sepsis (LOS), posing a threat to the lives of premature infants.

Therefore, researchers have actively explored effective preventive measures, with probiotics gaining attention as a potential strategy. Studies have revealed that the composition of the intestinal microbiota in preterm infants differs depending on their age and birth weight [5]. Preterm infants exhibit a reduction in bacterial diversity and a different gut microbiota, with more Proteobacteria and Enterococcus, which are considered potential pathogenic bacteria within the intestinal tract [6]. Targeted supplementation with probiotics and/or prebiotics has been shown to enhance the intestinal mucosal barrier function in preterm infants and competitively inhibit the growth of gastrointestinal pathogenic bacteria [7]. However, the effects of different probiotic genera or combinations may vary based on their morphological, physiological, and biochemical characteristics, as well as their interactions. Consequently, further research is required to determine the optimal probiotic supplementation protocol.

To address this issue, our study employed the network meta-analysis (NMA) to compare the effectiveness of different types of probiotics, either used alone or in combination, in preventing NEC and other related outcomes in preterm infants. Our advantage lies in the use of NMA with a Bayesian approach, assisted by the R software BUGSnet package [8, 9]. This allows us to obtain the posterior probability distribution of all relative intervention treatment effects, enabling us to estimate the relative intervention effects and quantify the uncertainty of parameter estimation. Compared to traditional meta-analysis, NMA also integrates indirect evidence that has not been directly compared to rank the clinical efficacy or harms of a series of interventions in a specific disease area. This can aid in the development of clinical guidelines, optimize decision-making processes, and analyze cost-effectiveness.

## Methods

### Search strategy and selection criteria

This Bayesian NMA adhered to the guidelines of the Cochrane Neonatal Review Group (https://neonatal.cochrane.org) and the Preferred Reporting Items for Systematic Reviews and Meta-Analyses (PRISMA) guidelines [10]. Two authors conducted an extensive search for randomized controlled trials in PubMed, Cochrane Library, Web of Science, Embase, and OVID databases using EndnoteX9. The search encompassed all records from the inception of each database until August 12, 2024. A detailed search formula can be found in the Table S1.

The inclusion criteria for this study were as follows: (i) a randomized controlled study with complete information and comparable intervention and control groups; (ii) the intervention was the addition of a single- or multi-strain probiotic; (iii) the study was conducted on preterm infants whose parents provided informed consent; (iv) at least one of the primary outcome indicators selected for this article was reported; and (v) the language of the article was English.

The exclusion criteria for the study were as follows: (i) duplicate published datasets; (ii) inclusion of preterm infants with serious conditions such as congenital gastrointestinal malformations, or death prior to the establishment of minimal enteral feeding; (iii) studies that included both term and preterm infants were only considered if data on preterm infants were reported separately.

Two researchers independently reviewed the title, abstract, and full text of the article to determine if it met the inclusion criteria. Any uncertainties were resolved by discussion with a third author.

### Data extraction

The characteristics of the studies included in the analysis comprised of the first author, year of publication, country of study, inclusion criteria, sample size, intervention, and primary outcome. Additionally, the characteristics of the study population included sex, gestational age, birth weight, mode of delivery, presence of multiple births, 5-minute Apgar score, type of feeding, use of prenatal glucocorticoids and postnatal antibiotics, time of initial feeding, and duration of total parenteral nutrition, are listed in Table S2. The interventions were categorized into 15 broad categories, as shown in Table S3.

### Outcome definition

During the design phase of our study, we identified a lack of consistent diagnostic criteria for FI and a dearth of new research on the incidence of FI [11]. As a result, the primary outcomes of the study, which were all determined before discharge from hospital, included all-cause mortality, incidence of NEC (at or above Bell’s stage II), mean length of hospitalization, time to achieve full enteral feeding, and incidence of sepsis with positive blood cultures (refer to Table S4). The modified Bell’s staging criteria were based on the degree of NEC progression, as indicated by the children’s clinical indicators and abdominal X-rays. Stage II was identified as the confirmed stage of NEC, which was characterized by the onset of systemic symptoms and abdominal signs, as well as the gradual emergence of limited peritonitis [12]. Efforts were made to contact the corresponding author of articles with missing data, but unfortunately, no response was received. In cases where only the median, range, and sample size were available for continuous results, we estimated the mean and standard deviation using the methods described in the book “Systematic Evaluation, Meta-Analysis Design, and Implementation Methods” edited by Liu et al.

### Quality of evidence

The risk of bias in the included studies was independently evaluated by two investigators using the Cochrane Risk of Bias tool within Review Manager 5.4. The review process focused on key factors, including randomized sequence generation, allocation concealment, blinding of study participants and healthcare providers, blinding of outcome assessors, incomplete outcome data, selective reporting, and other sources of bias. The ratings for each of these seven criteria were categorized into three primary options: low, high, and unclear.

### Statistical analysis

A Bayesian theorem with Markov chain Monte Carlo (MCMC) simulations using the BUGSnet software package, version R 4.4.1, was used to perform an NMA of the primary outcomes. The MCMC simulations consisted of 1000 iterations each, with a total of 10,000 iterations. Leverage plots were used to select fixed- or random-effects models, and the convergence of the models was verified through trajectory and density plots. Inconsistency between direct and indirect evidence was assessed by constructing inconsistency models. Publication bias was assessed using funnel plots (Figure S1). Additionally, we considered whether the studies were preregistered and if there was selective reporting of results.

In this study, the parameters in the NMA model were estimated and compared to other treatment measures. The likelihood function was dependent on the outcome type. Dichotomous outcomes were reported as risk ratio (RR) with 95% confidence intervals [95% CIs]. For continuous outcomes, the results were presented as weighted mean differences (MDs) with corresponding 95% CIs. A bilateral p-value less than 0.05 was considered statistically significant. The interventions were ranked using the Surface Under the Cumulative Ranking Curve (SUCRA) probability. The larger the surface under the curve, the higher the ranking of the intervention.

We performed a conventional meta-analysis of two direct comparisons using Review Manager 5.4, examining heterogeneity by calculating I^2^ statistics. Additionally, we conducted an NMA using the frequency theory approach in STATA17 to assess the sensitivity of the Bayesian approach results through comparison.

## Results

**Fig. 1:**
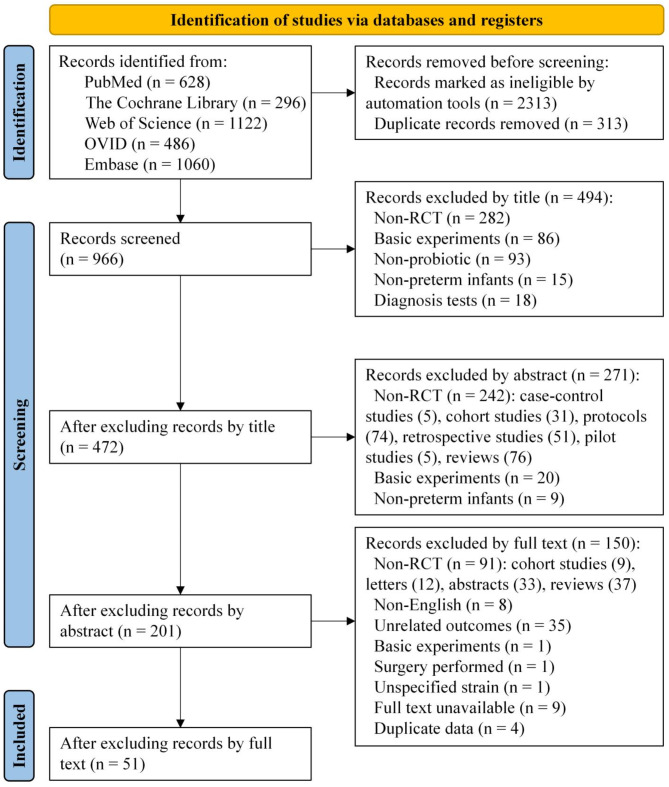
Flow diagram for searching and selecting eligible studies. The diagram shows the number of records identified, included, and excluded, along with the reasons for exclusions

**Fig. 2:**
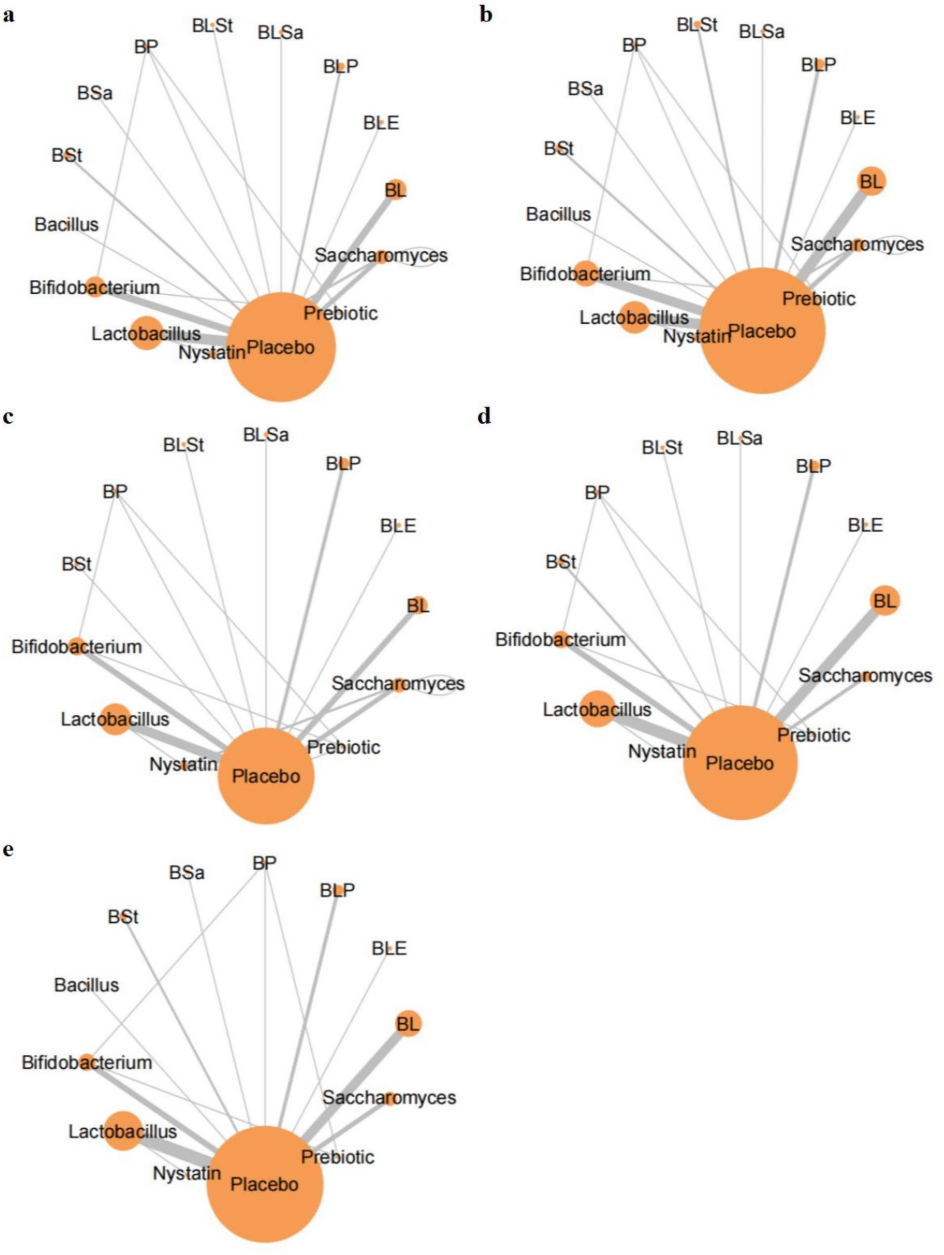
Network plot. This figure displays the network of eligible comparisons for (**A**) mortality, (**B**) incidence of necrotizing enterocolitis (at or beyond Bell Stage II), (**C**) length of hospital stay, (**D**) time to reach full feeding, and (**E**) incidence of culture-confirmed sepsis. The size of the nodes corresponds to the number of patients assigned to the intervention group, while the thickness of the lines corresponds to the number of direct comparison trials. Abbreviations: BL, Bifidobacterium + Lactobacillus; BLP, Bifidobacterium + Lactobacillus + Prebiotic; BSa, Bacillus + Saccharomyces boulardii; BSt, Bacillus + Streptococcus; BLE, Bifidobacterium + Lactobacillus + Enterococcus; BLSa, Bifidobacterium + Lactobacillus + Saccharomyces boulardii; BLSt, Bifidobacterium + Lactobacillus + Streptococcus; BP, Bifidobacterium + Prebiotic

A total of 51 randomized controlled studies were included in the analysis, involving data on 11,661 newborns from 19 countries [13–63] (Fig. 1). We then mapped the network evidence for all primary outcomes (Fig. 2). Fifteen of the included studies reported exclusive breastfeeding as the feeding method of the subjects [13, 18–21, 26, 28, 32–35, 39, 41, 44, 55], while two study reported formula feeding [14, 58]. Twenty-six studies reported mixed feeding. The remaining eight studies did not mention the feeding method [22, 30, 42, 45, 46, 54, 56, 62]. However, owing to the limited data available, we did not conduct further comparative analyses of economic levels and breast milk volume between the intervention and control groups.

All studies, except for two that used nystatin only as an intervention in the control group [49, 56], included blank or placebo controls. Most studies initiated the intervention during the initial feeding phase, on the second or third day of life for preterm infants, administering it once a day until hospital discharge. The intervention was administered orally or through tube feeding, with dosages ranging from 10^6^ to 10^10^ CFU. One particular study examined the impact of different dose levels and treatment durations on the efficacy of the probiotic [53]. In the remaining studies, the treatment course lasted more than 7 days (Table 1).

**Table 1:** Characteristics of the included studies

Study	Country	Sample size	Control	Intervention (Daily dosage)	Length of intervention(from/to)		Outcomes				
Taciana D 2011	USA	231	blank	B.breve + L.casei (3.5 × 10^7^ to 3.5 × 10^9^ CFU)	2 days	29 days	1	2		4	
Lingfen X 2016	China	125	blank	S.boulardii CNCM I-745 (10^9^ CFU twice daily)	< 7 days	> 7 days or discharge	1	2	3		5
ALONA 2005	Israel	145	blank	B.infantis + S.thermophilus + B.bifidus (each 0.35 × 10^9^ CFU)	feeding starts	36 weeks postconceptual age	1	2		4	5
Dilek D 2015	Turkey	400	placebo	B.lactis (5 × 10^9^ CFU)	2–3 days	discharge	1	2	3	4	5
				prebiotic (inulin, 900 mg)							
				B.lactis (5 × 109 CFU) + prebiotic (inulin, 900 mg)							
Kate C 2016	UK	1315	placebo	B.breve BBG-001 (8.2 to 9.2 log_10_ CFU)	as soon as possible	36 weeks postmenstrual age or discharge	1	2	3	4	5
Stephane H 2015	France	199	placebo	B.lactis (1 × 10^9^ CFU)	1 week	4/6 weeks		2			
				B.longum (1 × 10^9^ CFU)							
				B.lactis + B.longum (1 × 10^9^ CFU)							
Costalos 2003	Greece	87	placebo	S.boulardii (10^9^ CFU twice daily)	3.2 days	30 days				4	5
Manzoni 2006	Italy	80	blank	L.rhamnosus (6 × 10^9^ CFU)	3 days	6 weeks or discharge		2	3	4	5
Shashidhar 2017	India	104	blank	L.acidophilus + L.rhamnosus + B.longum + S.boulardii (1.25 × 10^9^ CFU)	2 days	mean 26.3 days	1	2	3	4	
Elaheh A 2017	Iran	60	blank	S.thermophilus + L.rhamnosus + L.acidophilus + L.bulgaricus + B.infantis + L.casei. (1 × 10^9^ CFU)		13.2 days		2			
Carlo D 2002	Italy	585	placebo	L.rhamnosus GG (6 × 10^9^ CFU)	feeding starts	discharge		2			5
W.A.M 2010	Germany	183	placebo	B.lactis 12 × 10^9^ CFU/kg/day	feeding starts	7–35 days	1	2		4	
M.A.H 2012	USA	101	blank	L.rhamnosus GG + B.infantis (each species 500 million CFU)	the first enteral feeding	34 weeks postmenstrual age or discharge	1	2			5
G.A.J 2022	Australia	173	REF group(PANTS)	B.breve M-16 V (3 × 10^9^ CFU)	mean 3 days	discharge	1	2			5
				B.breve M-16 V + B.longum subsp. infantis M-63 + B.longum subsp. longum BB536 (each species 1 × 10^9^ CFU)							
O.S.P 2020	Turkey	248	placebo	820 million L.rhamnosus + 410 million L.plantarum + 410 million L.casei + 410 million B.lactis + 383 mg fructooligosaccharide + 100 mg galactooligosaccharide + 2 mg bovine lactoferrin + 25 mg vitamin C + 8 mg vitamin E + 0.5 mg vitamin B1, B2 and B6	feeding starts	discharge	1	2	3		5
Chowdhury 2016	Bangladesh	120	blank	Bifidobacterium spp. + Lactobacillus (each species 3 × 10^9^ CFU)	feeding starts	discharge, at least 10 days		2	3	4	
Gamze D 2013	Turkey	271	blank	S.boulardii (5 billion CFU)	feeding starts	discharge	1	2	3	4	5
S Dongol Singh S 2017	Nepal	72	blank	L.rhamnosus 35 (0.8 mg in infants > 1500 g and 0.4 mg in infants < 1500 g in 2 ml of expressed breast milk two times daily)	3 days	30 days	1	2			
Fernández 2013	Mexico	150	blank	L.acidophilus (1.0 × 10^9^ CFU/g) + L.rhamnosus (4.4 × 10^8^ CFU/g) + L.casei (1.0 × 10^9^ CFU/g) + L.plantarum (1.76 × 10^8^ CFU/g) + B.infantis (2.76 × 10^7^ CFU/g) + S.thermophillus (6.6 × 10^5^ CFU/g)	median 5 days	median 38 days	1	2	3	4	
Moumita S 2009	India	186	blank	B.infantis + Bifidobacteria bifidum + B.longum + L.acidophilus (2.5 billion CFU twice daily)	mean 5.97 days	discharge	1	2	3	4	5
E.V.N 2015	South Africa	184	HIV-unexposed placebo	HIV-unexposed L.rhamnosus + B.infantis (each species 0.35 × 10^9^ CFU)	feeding starts	28 days		2		4	5
				HIV-exposed Placebo							
				HIV-exposed L.rhamnosus GG + B.infantis (each species 0.35 × 10^9^ CFU)							
V.V.T 2015	India	244	Very preterm placebo	Very preterm Bacillus clausii (2.4 × 10^9^ CFU)	day 5 in asymptomatic and day 10 in symptomatic neonates	6 weeks or discharge	1	2			5
				Extreme preterm placebo							
				Extreme preterm Bacillus clausii (2.4 × 10^9^ CFU)							
Havranek 2013	USA	31	placebo	L.rhamnosus + B.infantis (each species 500 million CFU)	mean 9.4 days	34 weeks or discharge				4	
M Strus 2018	Poland	181	placebo	L.rhamnosus KL53A + B.breve PB04 (1 × 10^6^ CFU)		6 weeks or discharge	1	2			5
Susan E 2013	Australia	1099	placebo	B.infantis + S.thermophilus + B.lactis (1 × 10^9^ CFU)	mean 5 days	40 weeks or discharge	1	2	3	4	5
Risma K 2019	Indonesia	94	placebo	L.reuteri DSM 17,938 (1 × 10^8^ CFU)	feeding starts	discharge, at least 7 days	1	2	3	4	5
H.C.L 2005	China	367	blank	L.acidophilus + B.infantis (each species 1 × 10^9^ CFU, twice daily)	mean 7.7 days	discharge		2			5
H.C.L 2008	China	443	blank	L.acidophilus + B.infantis (each species 10^9^ CFU, twice daily)	mean 4.5 days	6 weeks	1	2	3	4	5
Erik W 2018	Sweden	134	placebo	L.reuteri DSM 17,938 (1.25 × 10^8^ CFU)	< 3 days	gestational week 36 + 0	1	2		4	5
Mazyar V 2020	Iran	106	placebo	B.infantis + Lactobacillus rhamnosus + L.reuteri + fructooligosaccharide (2.5 × 10^8^ CFU)	1.83 ± 0.580 days	discharge			3	4	
Belal A 2022	Canada	62	blank	B.breve HA-129 + B.bifidum HA-132 + B.longum subsp + infantis HA-116 + B.longum subsp + longum HA-135 + L.rhamnosus HA-111 (4 billion CFU)		37 weeks corrected gestational age/discharge			3	4	5
L.P.N 2015	India	220	blank	L.acidophilus (700 million CFU) + B.longum (400 million CFU) + L.rhamnosus (400 million CFU) + L.plantaris (300 million CFU) + L.casei (300 million CFU) + L.bulgaricus (300 million CFU) + B.infantis (300 million CFU) + B.breve (300 million CFU) + 100 mg fructooligosaccharide	feeding starts			2	3	4	5
Satsuki T 2014	Japan	283	placebo	B.bifidum OLB6378 (2.5 × 10^9^ CFU)	< 21 days		1	2	3	4	5
Flavia I 2017	Italy	60	placebo	L.reuteri DSM 17,938 (1 × 10^8^ CFU)	48 h	30 days			3	4	
Marwyn S 2022	South Africa	200	placebo	L.acidophilus + B.bifidum + B.infantis (each species 0.67 billion CFUs)		28 days				4	
M.Y.O 2014	Turkey	424	placebo	L.reuteri DSM 17,938 (1 × 10^8^ CFU)	feeding starts	discharge	1	2	3	4	5
M.Y.O 2015	Turkey	316	nystatin (100,000 U/ml every 8 h)	L.reuteri DSM 17,938 (1 × 10^8^ CFU)	feeding starts	discharge	1	2	3	4	5
İpek Güney V 2017	Turkey	119	blank	L.rhamnosus (4.1 × 10⁸ CFU) + L.casei (8.2 × 10⁸ CFU) + L.plantorum (4.1 × 10⁸ CFU) + B.animalis (4.1 × 10⁸ CFU) + 383 mg ructooligosaccharides + 100 mg galactooligosaccharides	feeding starts	discharge	1	2		4	5
M.A.R 2012	USA	750	blank	L.reuteri DSM 17,938 (1 × 10^8^ CFU)	as early as possible	discharge	1	2	3		5
Sanjay P 2014	Australia	159	placebo	B.breve M-16 V (3 × 10^9^ CFU)	feeding starts	corrected age of 37 weeks	1	2	3	4	5
Sourabh D 2015	India	149	placebo	high dose, long course (10 × 10^9^ CFU)	72 h	long course 21 days, short course 1–14 days	1	2			5
				high dose, short course (10 × 10^9^ CFU)	72 h						
				low dose, long course (1 × 10^9^ CFU)	93.5 h						
Ghasem B 2021	Iran	76	placebo	B.lactis (1 × 10^9^ CFU)	< 24 h			2	3		
Mahtab M 2022	Iran	78	placebo	Probiotic-mothers: L.paracasei (1.5 × 10^9^ CFU/g)		28 days	1		3	4	5
				Probiotic-infants: L.paracasei (1.5 × 10^9^ CFU/g)							
Gamze D 2013	Turkey	181	nystatin (100,000 U/ml every 8 h)	S. boulardii (5 × 10^9^ CFU)	feeding starts	discharge	1	2	3		
Zlatka K 2015	Slovenia	80	blank	L.acidophilus + E.faecium + B.infantum (1.5:1:1.5, 0.6 × 10^7^ CFU twice daily)			1	2	3	4	5
Xuewei C 2019	China	114	blank	L.reuteri DSM 17,938 (1 × 10^8^ CFU)	feeding starts	discharge			3	4	5
Varaporn S 2014	Thailand	60	blank	L.acidophilus + B.bifidum (1 × 10^9^ CFU, twice a day)	feeding starts	6 weeks or discharge	1	2	3	4	5
FN Sari 2011	Turkey	221	blank	L.sporogenes (3.5 × 10^8^ CFU)	feeding starts	discharge	1	2		4	5
Ozge S 2013	Turkey	208	placebo	S.boulardii (0.5 × 10^9^ cell/kg, twice daily)	feeding starts	discharge	1	2	3	4	5
M.N.S 2015	Iran	60	blank	L.reuteri DSM 17,938 (20 million/kg every 12 h)	mean 3.2 days	feeding at 120 ml/kg per day	1			4	5
Manish Rasania 2023	India	123	blank	L.rhamnosus GG 6 × 10^9^ CFU	feeding starts	4 weeks or corrected age of 36 weeks	1	2	3	4	5
				L.rhamnosus GG 2 × 10^9^ CFU							

The study’s first author, publication date, region, study population, intervention, and primary outcomes are described. Primary outcome 1 was mortality; primary outcome 2 was the incidence of necrotizing enterocolitis (at or beyond Bell Stage II); primary outcome 3 was the length of hospital stay; primary outcome 4 was the time to reach full feeding; primary outcome 5 was the incidence of culture-proven sepsis

The network evidence plots indicated that all interventions, except nystatin, were directly compared with the placebo at least once. We conducted a Bayesian-framed random-effects NMA under the consistency model based on the results of the leverage plot and inconsistency model. The trajectory and density plots indicated relatively low variability among the included studies, suggesting similarity in the study design or characteristics of the study population.

**Fig. 3:**
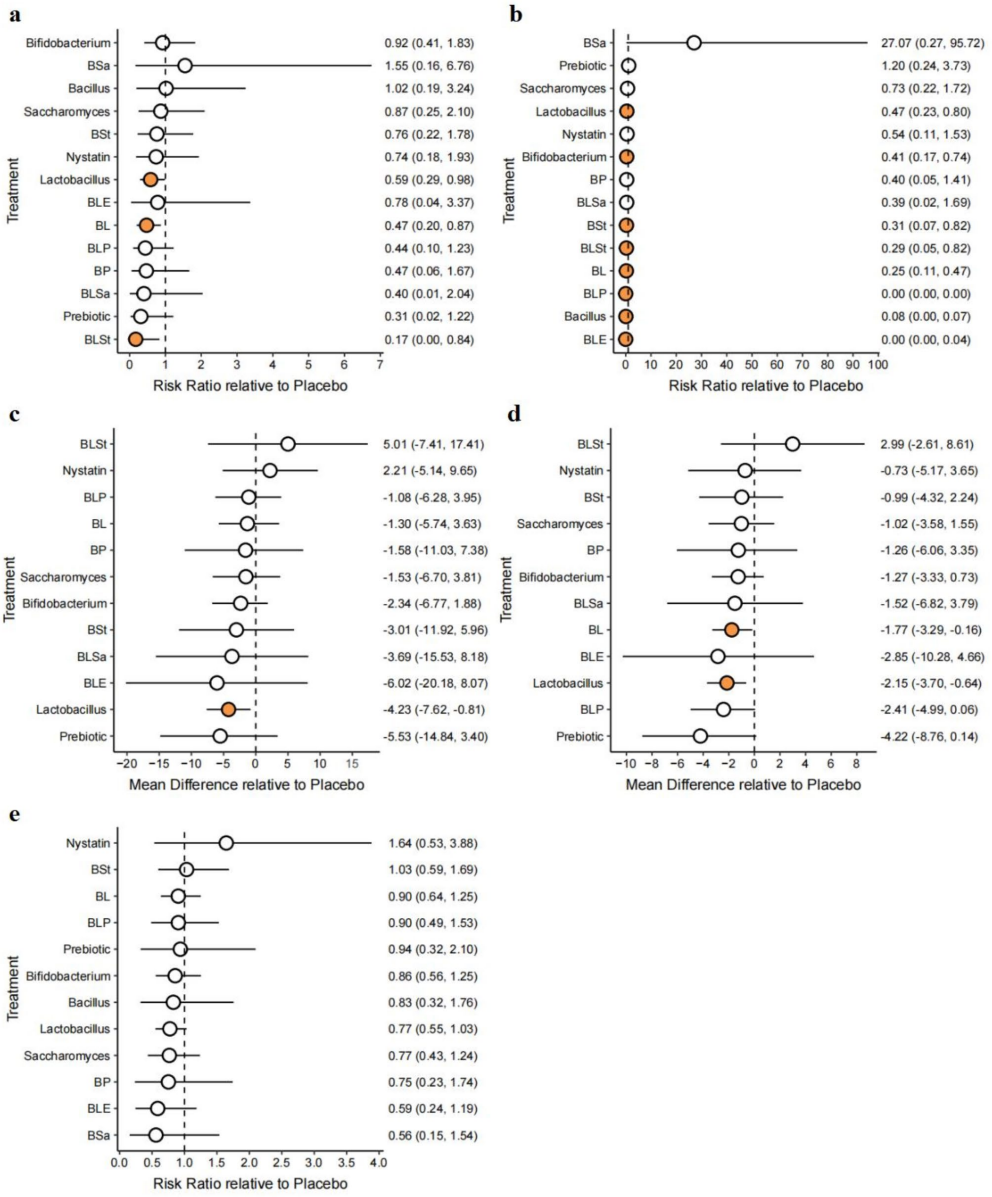
Forest plot with placebo as the common comparator. Summary effect estimates of the different interventions for (**A**) mortality, (**B**) incidence of necrotizing enterocolitis (at or beyond Bell Stage II), (**C**) length of hospital stay, (**D**) time to reach full feeding, and (**E**) incidence of culture-confirmed sepsis. The estimates display the posterior mean with a 95% confidence interval. Abbreviations: BL, Bifidobacterium + Lactobacillus; BLP, Bifidobacterium + Lactobacillus + Prebiotic; BSa, Bacillus + Saccharomyces boulardii; BSt, Bacillus + Streptococcus; BLE, Bifidobacterium + Lactobacillus + Enterococcus; BLSa, Bifidobacterium + Lactobacillus + Saccharomyces boulardii; BLSt, Bifidobacterium + Lactobacillus + Streptococcus; BP, Bifidobacterium + Prebiotic

The forest plot results (Fig. 3) indicated that prophylactic supplementation with probiotics and/or prebiotics showed potential or significant protection for preterm neonates. This protective effect was evident in the reduction of NEC incidence, which became even more significant when used in combination. Only Lactobacillus (RR, 0.59; 95% CI: 0.29, 0.98), BL (RR, 0.47; 95% CI: 0.20, 0.87) and BLSt (RR, 0.17; 95% CI: 0.00, 0.84) showed a significant decrease in all-cause mortality compared to the placebo. BL (MD, -1.77; 95% CI: -3.29, -0.16) and Lactobacillus (MD, -2.15; 95% CI: -3.70, -0.64) were effective in reducing the time taken for preterm infants to reach full enteral feeding. Additionally, Lactobacillus alone was effective in reducing the mean length of hospitalization in preterm infants (MD, -4.23; 95% CI: -7.62, -0.81). There is no evidence to suggest that probiotics either increase or decrease the risk of sepsis in premature infants. Finally, we rearranged the order of the individual interventions for the different primary outcomes in the forest plot according to the SUCRA probability.

Based on the results of the SUCRA plot (Figure S2) and the probability ranking plot (Figure S3), BLSt (RR, 0.17; 95% CI: 0.00, 0.84), the prebiotic (RR, 0.31; 95% CI: 0.02, 1.22), and BLSa (RR, 0.40; 95% CI: 0.01, 2.04) were the most effective interventions for reducing all-cause mortality during hospitalization in newborn preterm infants. The prebiotic and Lactobacillus were found to be very effective in reducing the time taken for preterm infants to stay in the hospital (MD of Prebiotic, -5.53, 95% CI: -14.84, 3.40) and reach full enteral feeding (MD of Prebiotic, -4.22, 95% CI: -8.76, 0.14). Consistent with the significant results of the forest plot, BLE (RR, 0.00; 95% CI: 0.00, 0.04), Bacillus (RR, 0.08; 95% CI: 0.00, 0.07) and BLP (RR, 0.00; 95% CI: 0.00, 0.00) ranked among the top three in reducing the incidence of NEC (at or above Bell stage II) in premature infants. To highlight the effect sizes, the league tables were visualized as a heat map (Figure S4).

The risk-of-bias summary and graph were presented in Figure S5. Funnel plots indicated no significant publication bias in the included studies. However, one study had a high risk of bias in terms of random sequence generation, allocation concealment, and blinding. Furthermore, although the majority of the studies did not provide clear sources of funding or blind the outcome evaluators, the risk of bias was minimal because of the objective nature of the selected evaluation metrics and the strong adherence exhibited by the preterm neonates. Consequently, the quality of the evidence from the studies included in this NMA was high.

To conduct a sensitivity assessment of the NMA, we compared its outcomes with those of conventional two-by-two direct comparison meta-analyses (Figure S6). By closely examining the MCMC trajectory plots and effect density plots, we identified five studies that potentially had an abnormal influence on the incidence of NEC. These studies used Bacillus [34], BLP [27, 44, 50], and BLE [57], as prophylactic measures against NEC, and none of their intervention groups (a total of 445 newborns) developed NEC (at or beyond Bell Stage II).

## Discussion

We conducted a systematic analysis of 51 randomized controlled studies to examine the effects of probiotics on NEC and other related outcomes in preterm infants. To exclude suspected cases, we selected NEC of Bell stage II or higher with more defined clinical features as the primary outcome. Our study confirmed a previous NMA, which suggests that only multiple-strain probiotics are associated with reduced all-cause mortality in preterm infants (RR, 0.69; 95% CI: 0.56, 0.86), while single-strain probiotics (MD, -1.94, 95% CI: -2.96, -0.92) and multi-strain probiotics (MD, -2.03, 95% CI: -3.04, -1.02) proved to be the most effective in reducing the time to reach full enteral feeding compared with placebo [64]. However, we further concluded that the best probiotic combination for reducing all-cause mortality in preterm infants was BLSt. Prebiotics and Lactobacillus were the most effective in reducing the length of hospitalization and the time to full enteral feeding in preterm infants.

Microbial diversity in the intestines of preterm infants usually decreases, the stability of the flora is reduced, and the number of harmful bacteria increases. Dysregulated microorganisms increase TLR4 signaling [65], ultimately resulting in cell death and intestinal mucosal barrier damage. Our analysis indicated that the combined use of Bifidobacterium, Lactobacillus, and Enterococcus was the most effective treatment in reducing the incidence of severe NEC, consistent with the results of a previous NMA study [66]. Previous reports have suggested that Bifidobacteria and Lactobacilli, when combined with other probiotics, can promote microbiome maturation and immune regulation in premature infants [67–69]. Bifidobacterium produces organic acids, antibacterial proteins, and H_2_O_2_, which help shape the intestinal microbial environment. On the other hand, Lactobacillus primarily enhances the intestinal barrier by inducing adhesion secretion and inhibiting cell apoptosis. Enterococcus faecalis possesses characteristics such as easy adhesion and fast growth, making it an ideal probiotic for effective functioning in the intestine [68, 70].

Multiple studies have shown that adding probiotics can reduce the duration of complete enteral feeding [66]. Compared to late-stage enteral feeding, early enteral feeding can reduce the incidence of FI, extrauterine growth restriction, and delayed sepsis in premature infants [71]. Recent randomized controlled trials have indicated that there is no significant association between the time it takes for preterm infants to reach full enteral feeding and their gestational age when monitoring gastric residues is not conducted [72]. This suggests that, in addition to incorporating probiotics, promoting early enteral feeding and minimizing unnecessary monitoring of gastric contents should be encouraged.

An extended hospital stay poses an increased risk for preterm infants [73]. Only Lactobacillus significantly decreased the duration of hospitalization in this study. Other studies have shown that Lactobacilli may enhance gastrointestinal movement, decrease gastroesophageal reflux, and facilitate weight gain in premature infants [58]. Furthermore, Lactobacilli can reduce the occurrence of ventilator-associated pneumonia through their anti-inflammatory properties [74].

Excessive probiotic consumption may raise safety concerns. Limited randomized controlled studies have strictly differentiated between early- and late-onset sepsis. Hence, this study adopted a uniform approach by defining sepsis as a positive blood culture. However, there is currently no evidence that probiotics cause serious adverse events in infants.

Given the potential impact of breast milk on the risk of NEC in preterm infants, future studies should prioritize the accurate quantification of breast milk volume in both intervention and control groups. Meanwhile, comparative studies between countries at different economic levels are essential to explore potential differences in the effectiveness of probiotic interventions. Addressing these research gaps will facilitate a more comprehensive understanding of the role of probiotics in the prevention of NEC in preterm infants and related outcomes. Furthermore, it will potentially lead to the development of more targeted and effective clinical strategies.

This study has several limitations, primarily its narrow focus on probiotic genera. Additionally, the inclusion criteria were limited to the English-language literature to ensure the quality of the studies analyzed. Due to the unavailability of individual-level data, the analysis could only be conducted at the pooled or study level. For the lack of response, several possible explanations are hypothesized. One possibility is that the corresponding authors are involved in other important research projects or have heavy workloads. Another factor could be that some of the emails were misdirected to their spam folders, or that their contact information has changed over time. In cases where the corresponding author was unable to provide the required data or did not respond to the email, the study was excluded from the meta-analysis.

## Conclusions

The combination of Bifidobacterium, Lactobacillus, and Enterococcus was the most effective in reducing the mortality and incidence of NEC (Bell II or above) in preterm infants. Lactobacillus may be the best option for reducing the length of hospitalization and time to full enteral feeding in preterm infants. There is no evidence to suggest that probiotics increase or decrease the risk of sepsis in preterm infants.

## Electronic supplementary material

Below is the link to the electronic supplementary material.

Supplementary Material 1

Supplementary Material 2

## Data Availability

All data generated or analysed during this study are included in this published article [and its supplementary information files].

## Abbreviations

FI: Feeding intolerance
LOS: Late-onset sepsis
NEC: Necrotizing enterocolitis
NMA: Network meta-analysis
BL: Bifidobacterium + Lactobacillus
BLP: Bifidobacterium + Lactobacillus + Prebiotic
BSa: Bacillus + Saccharomyces boulardii
BSt: Bacillus + Streptococcus
BLE: Bifidobacterium + Lactobacillus + Enterococcus
BLSa: Bifidobacterium + Lactobacillus + Saccharomyces boulardii
BLSt: Bifidobacterium + Lactobacillus + Streptococcus
BP: Bifidobacterium + Prebiotic
