# Supplementary material for: Effect of probiotics on necrotizing enterocolitis in preterm infants: a network meta-analysis of randomized controlled trials
Source: BMC Pediatr. 2025 Mar 27;25:237. doi: 10.1186/s12887-025-05469-z (PMC11948853; doi:10.1186/s12887-025-05469-z)
Supplement: Supplementary file 1 — Supplementary Material 1 [file 12887_2025_5469_MOESM1_ESM.docx]

Supplement figures

# Effect of Probiotics on Necrotizing Enterocolitis in Preterm Infants: A Network Meta-Analysis of Randomized Controlled Trials

Yu Dai^1,#^, Qinlei Yu^1,#^, Fan Zhang^1^, Ke Ma^1^, Xiangyun Yan^1^, Wenjuan Chen^1^, Xiaohui Chen^1,*^, Shushu Li^1,*^, Shuping Han^1,*^

^1^Department of Pediatrics, Women’s Hospital of Nanjing Medical University, Nanjing Women and Children’s Healthcare Hospital, Nanjing, China.

^#^ These authors contributed equally to this work.

**^*^** Corresponding author: shupinghan@njmu.edu.cn (S.P. Han), lishushu@njmu.edu.cn (S.S. Li), chenxiaohui@njmu.edu.cn (X.H. Chen)

**Figure S1: Funnel plot of comparison**

**Figure S2: SUCRA plot**

**Figure S3: Probability ranking diagram**

**Figure S4: League heat plot of the network estimates**

**Figure S5: Risk of bias assessment**

**Figure S6: Forest plot of two direct comparisons**

| **a** | **b** |
| --- | --- |
| **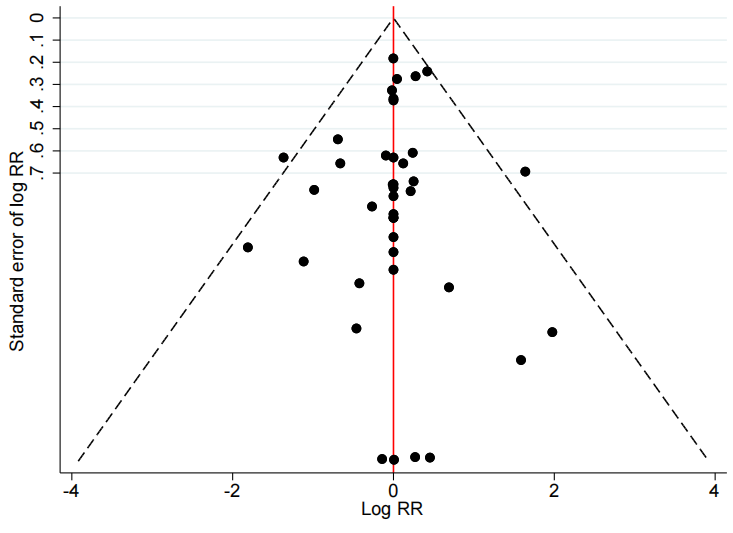** | **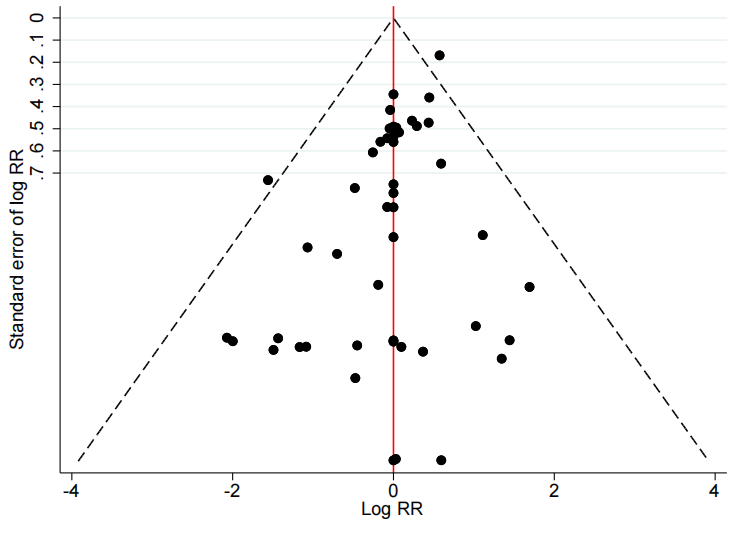** |
| **c** | **d** |
| **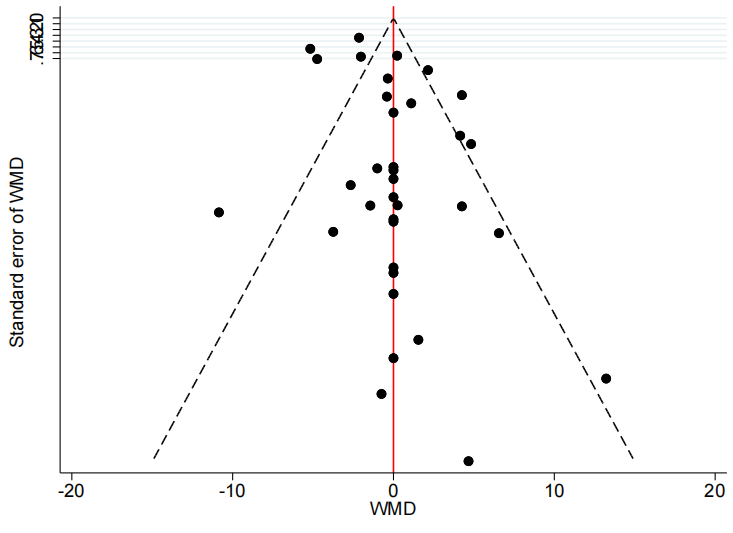** | **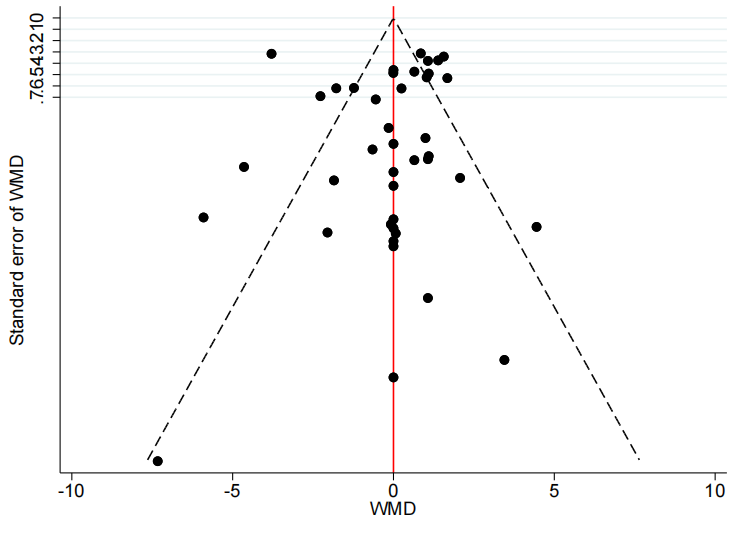** |
| **e** |  |
| 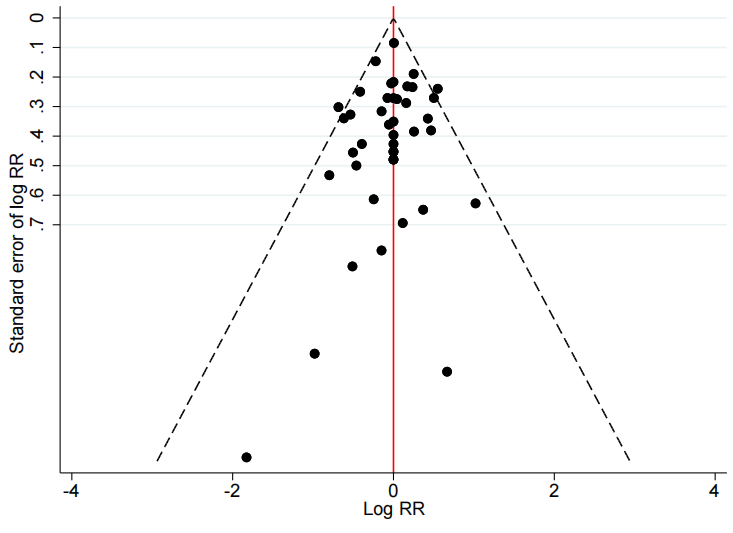 |  |

**Figure S1: Funnel plot of comparison.** Funnel plots are used to identify publication bias for (a) mortality, (b) incidence of necrotizing enterocolitis (at or beyond Bell Stage II), (c) length of hospital stay, (d) time to reach full feeding, and (e) incidence of culture-confirmed sepsis. The horizontal axis represents the effect size, while the vertical axis represents the inverse of the standard error of the logarithm of the effect size. The dashed lines on either side of the funnel plot indicate 95% confidence intervals.

| **a** | **b** |
| --- | --- |
| **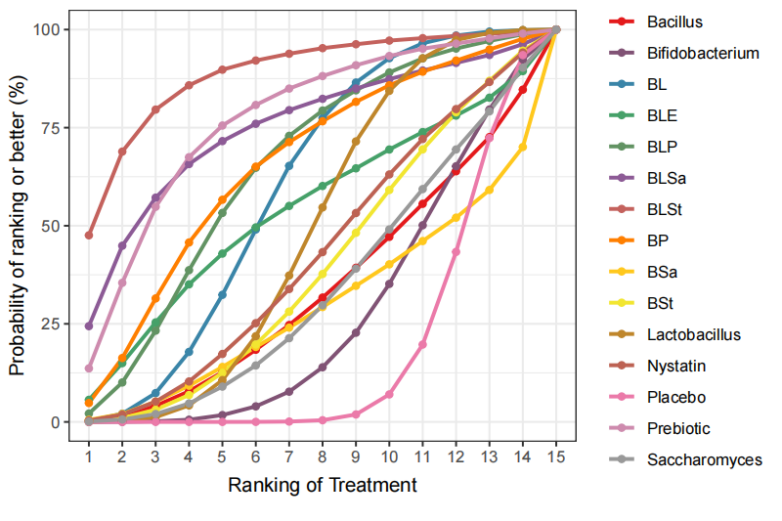** | **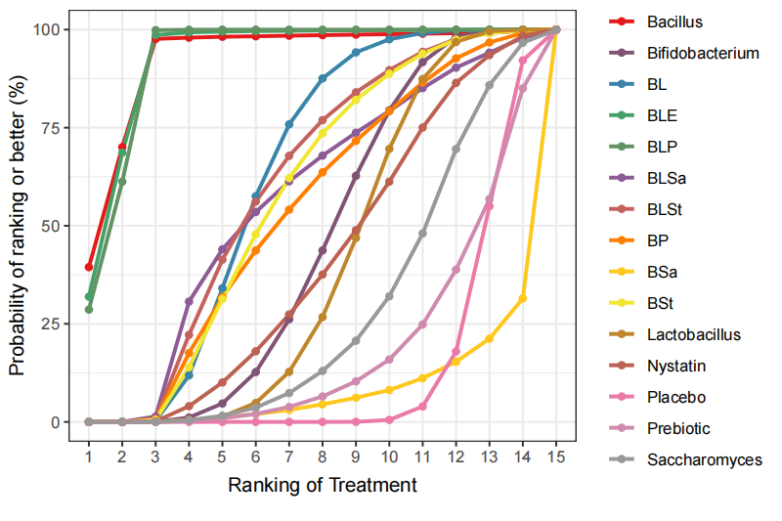** |
| **c** | **d** |
| **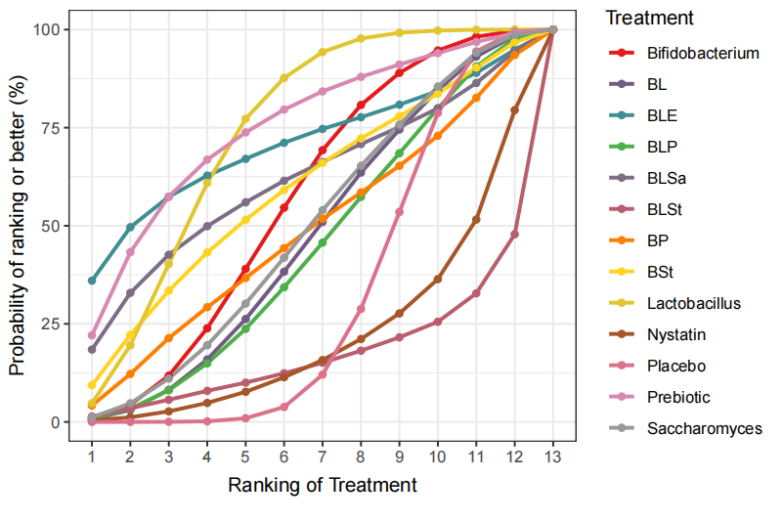** | **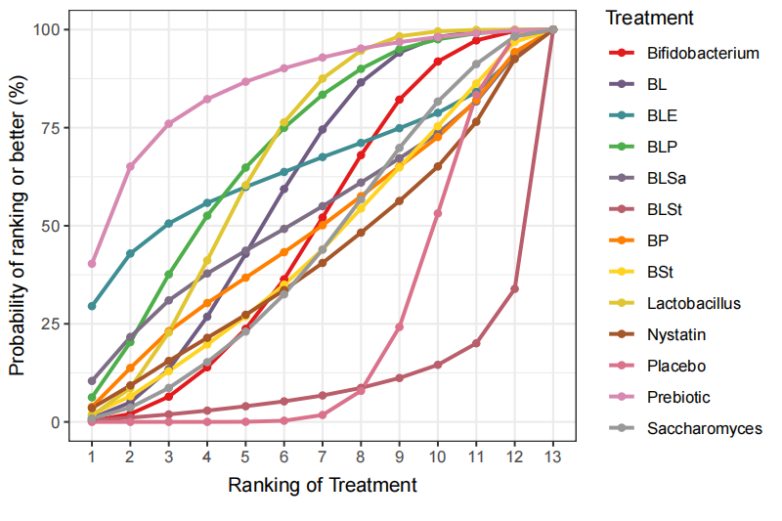** |
| **e** |  |
| **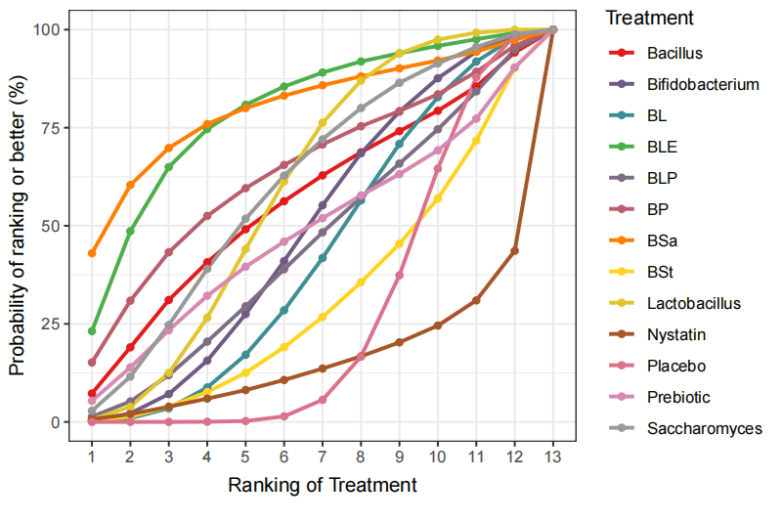** |  |

**Figure S2: SUCRA plot.** The surface area under the preferred probability ranking curve (SUCRA) about (a) mortality, (b) incidence of necrotizing enterocolitis (at or beyond Bell Stage II), (c) length of hospital stay, (d) time to reach full feeding, and (e) incidence of culture-confirmed sepsis. Higher rankings associated with smaller outcome values.

| **a** | **b** |
| --- | --- |
| 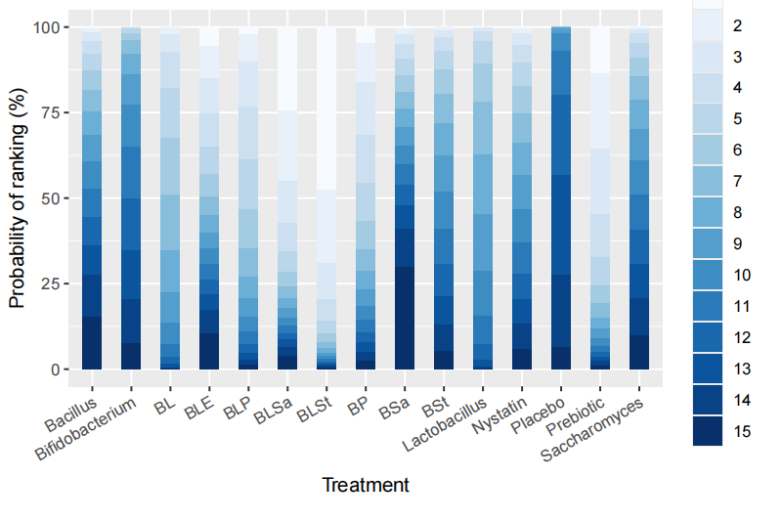 | 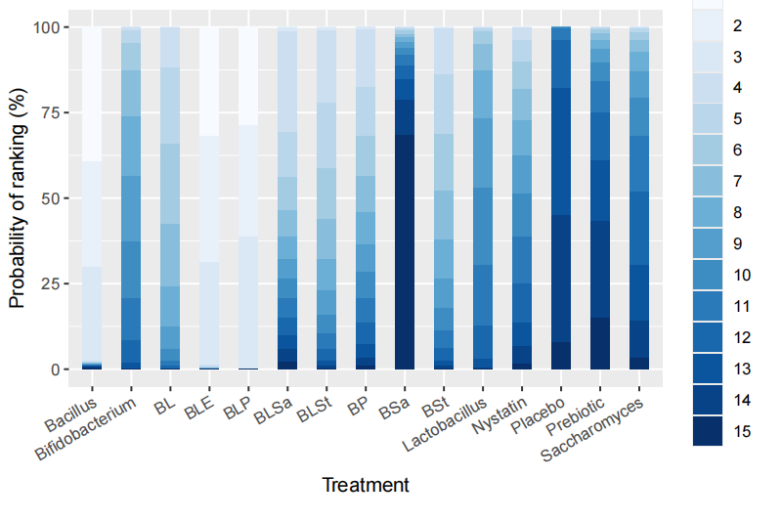 |
| **c** | **d** |
| 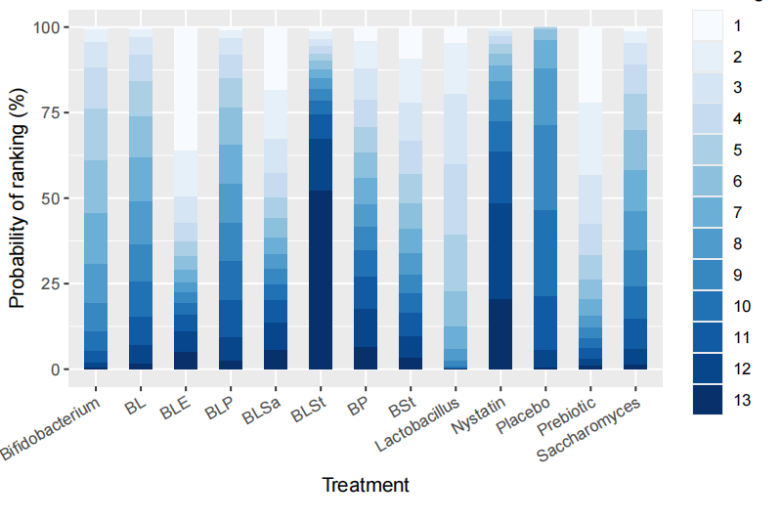 | 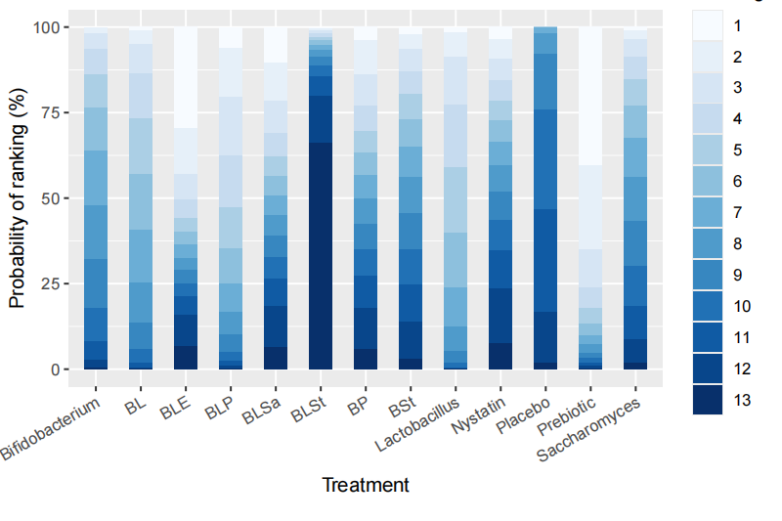 |
| **e** |  |
| 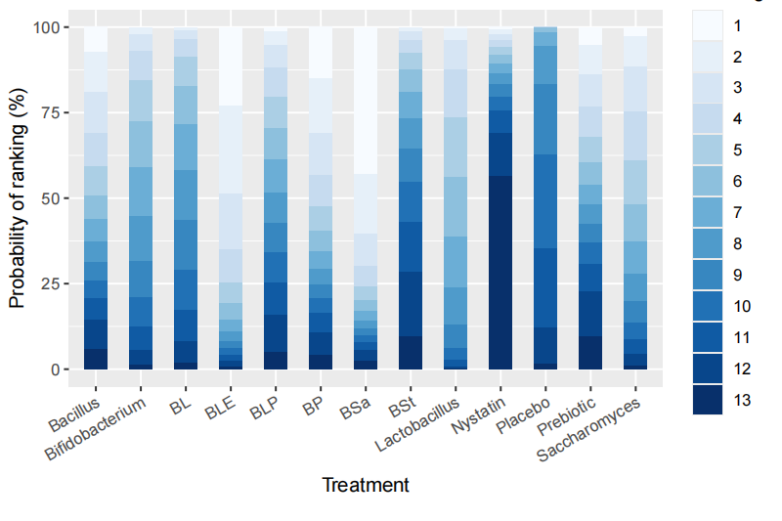 |  |

**Figure S3: Probability ranking diagram.** The interventions were ranked according to their effect sizes on (a) mortality, (b) incidence of necrotizing enterocolitis (at or beyond Bell Stage II), (c) length of hospital stay, (d) time to reach full feeding, and (e) incidence of culture-confirmed sepsis. Smaller effect sizes indicate better results. Higher rankings were associated with smaller outcome values.

**a**


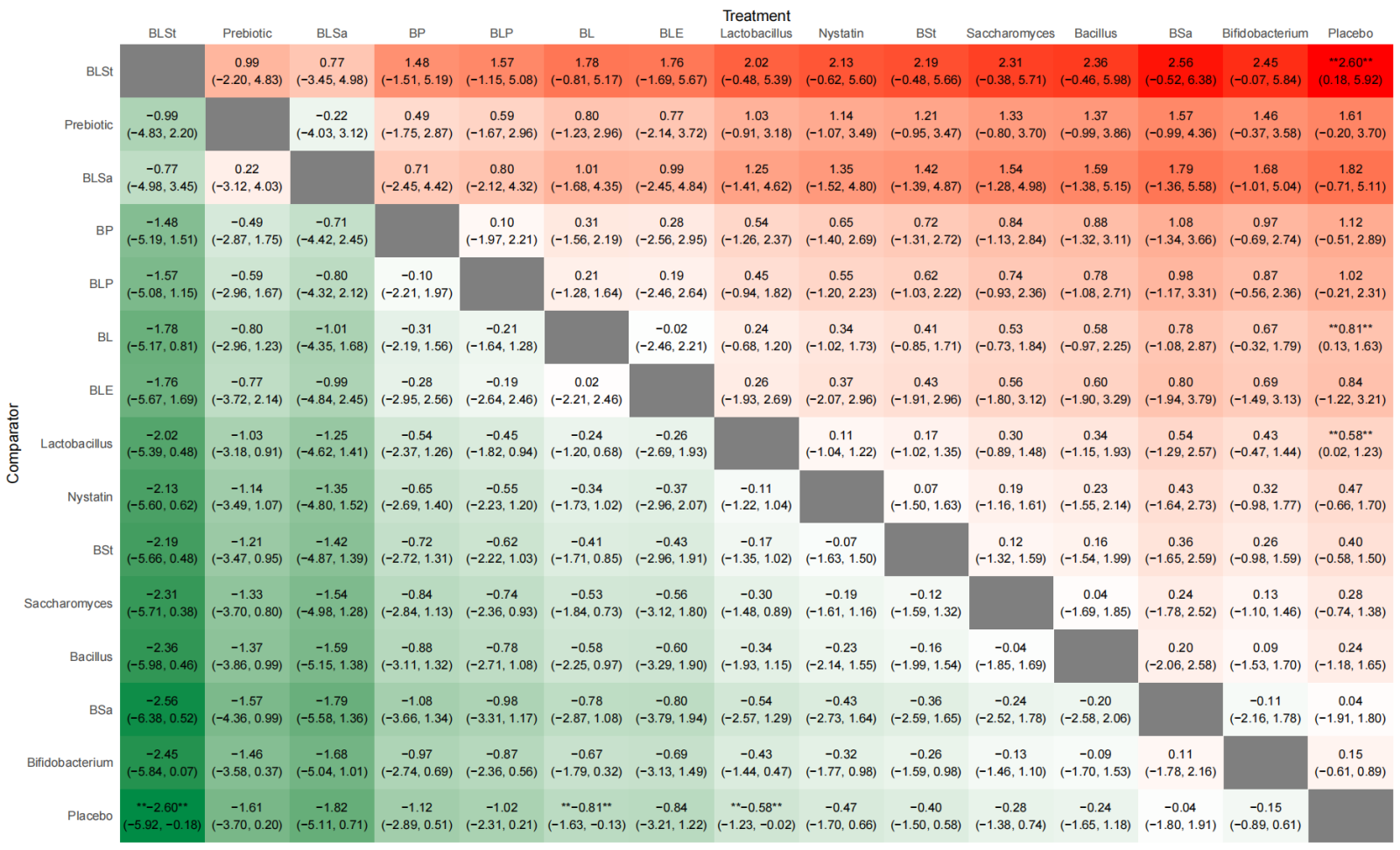


**b**


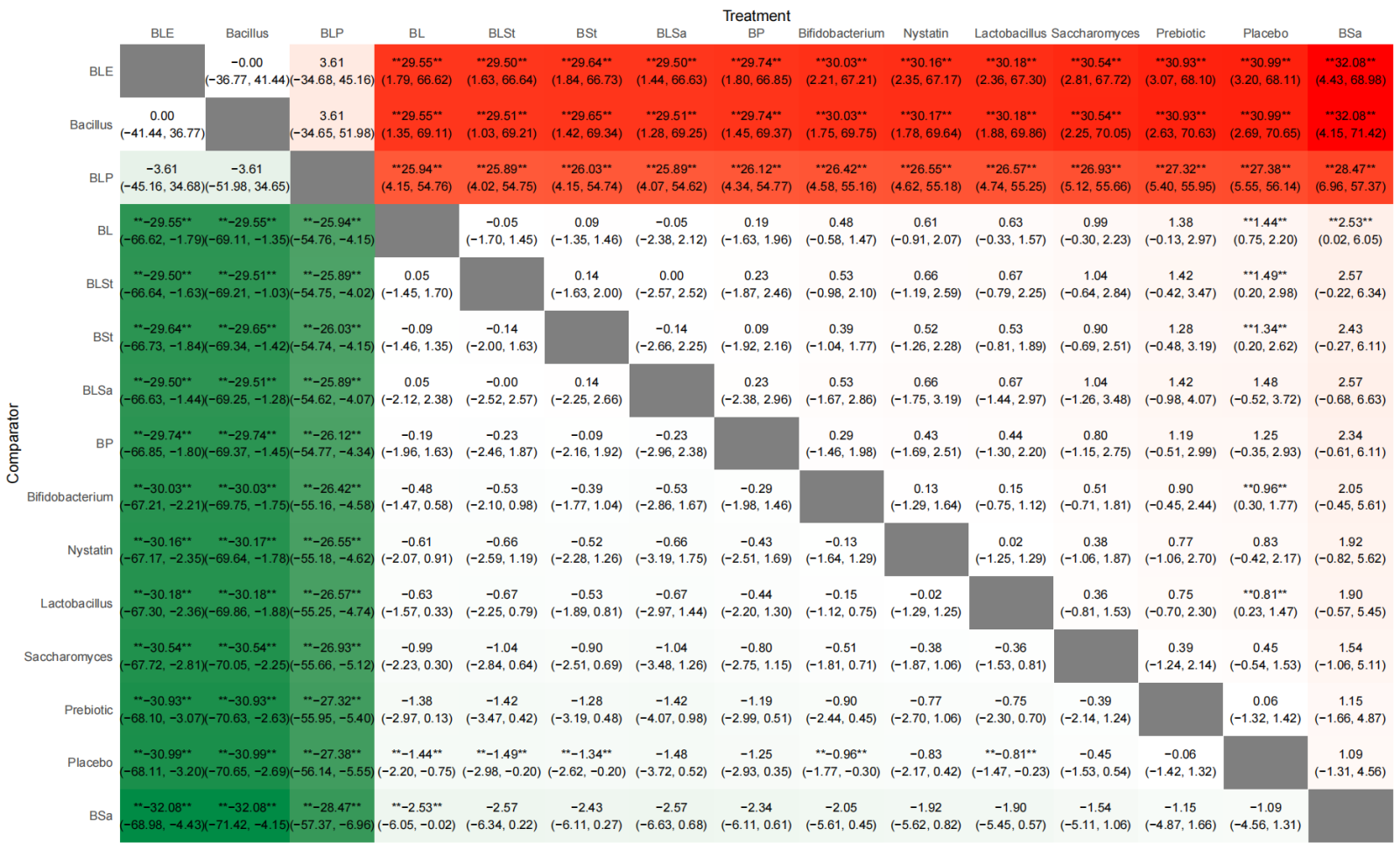


**c**


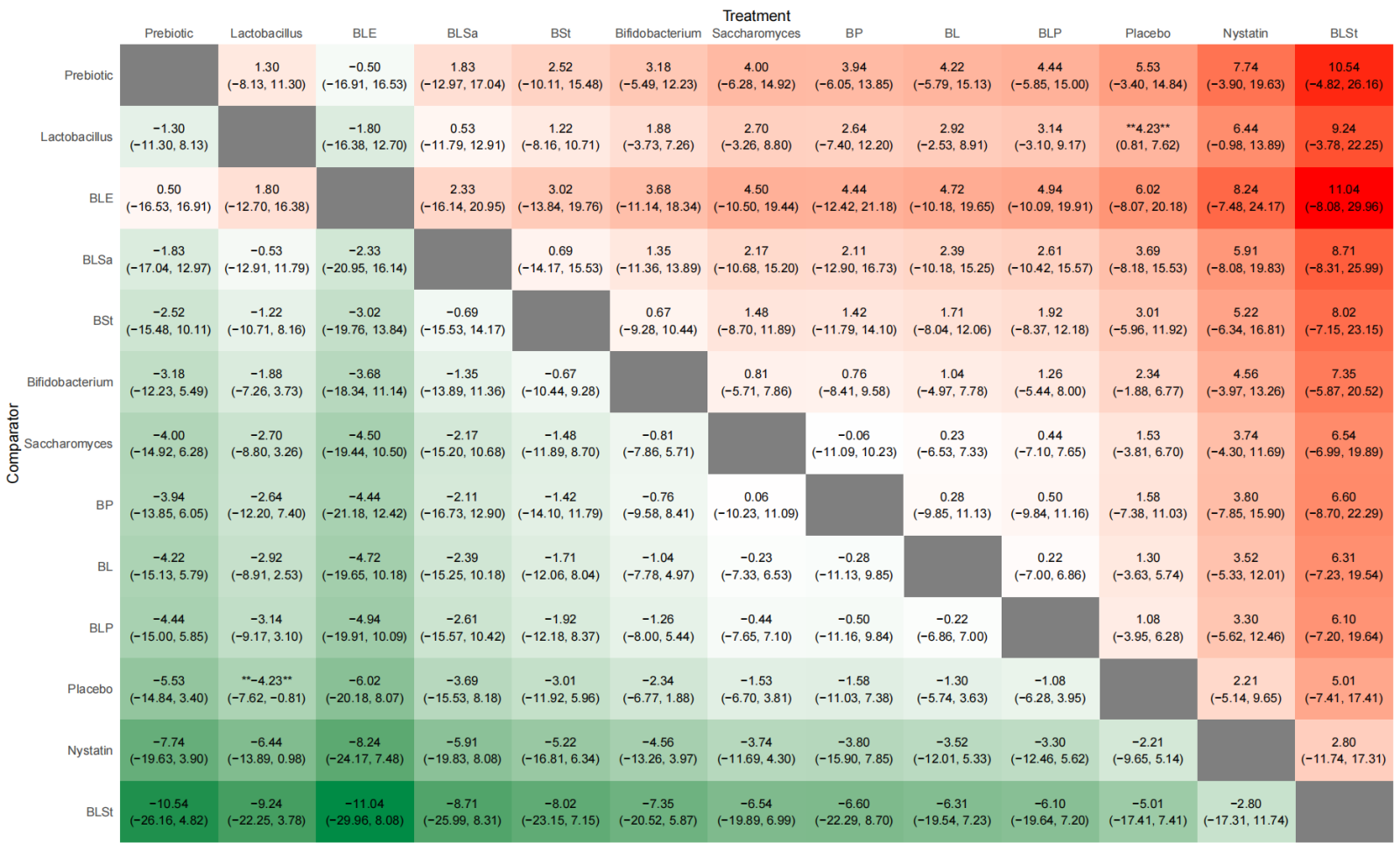


**d**


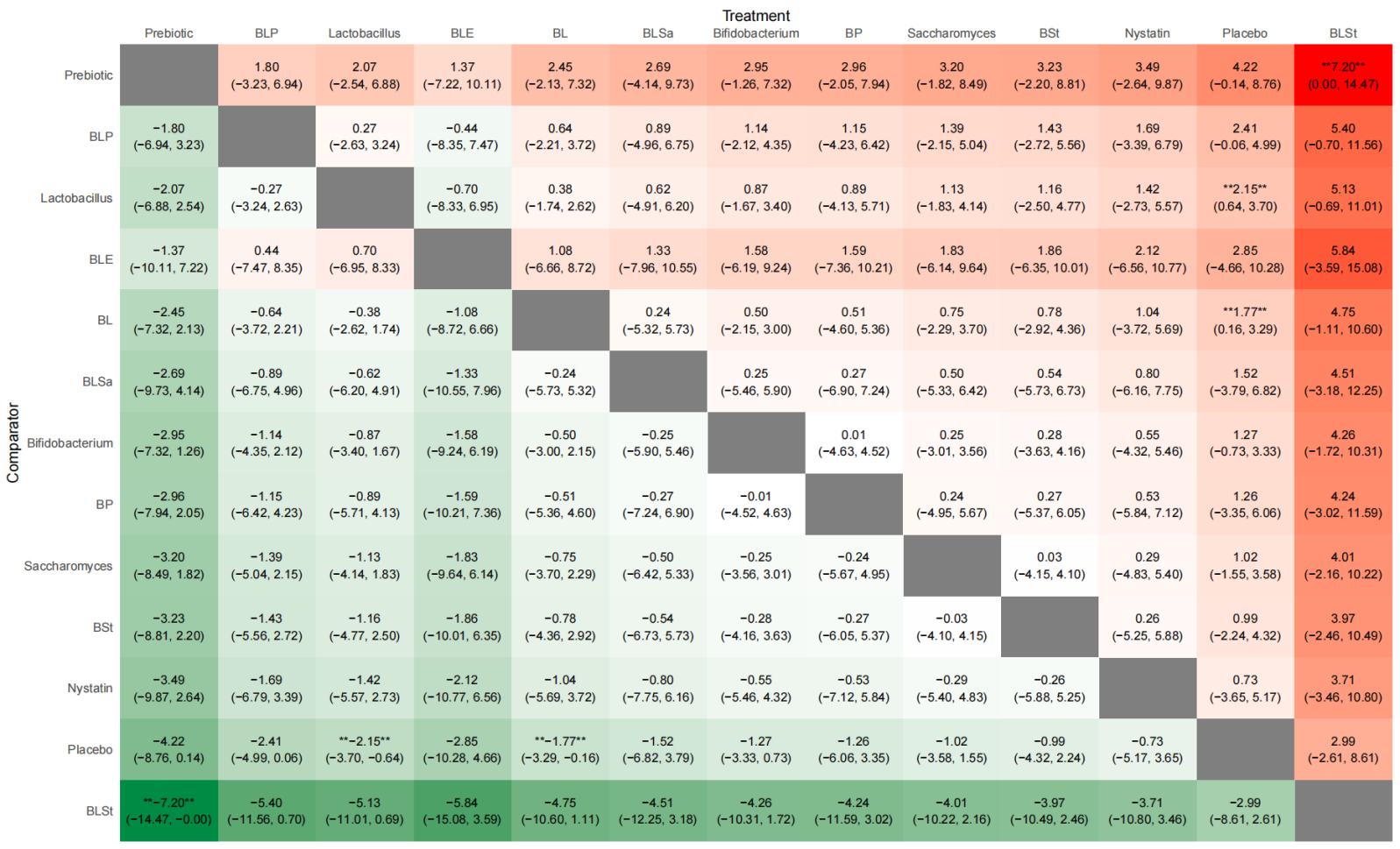


**e**

**
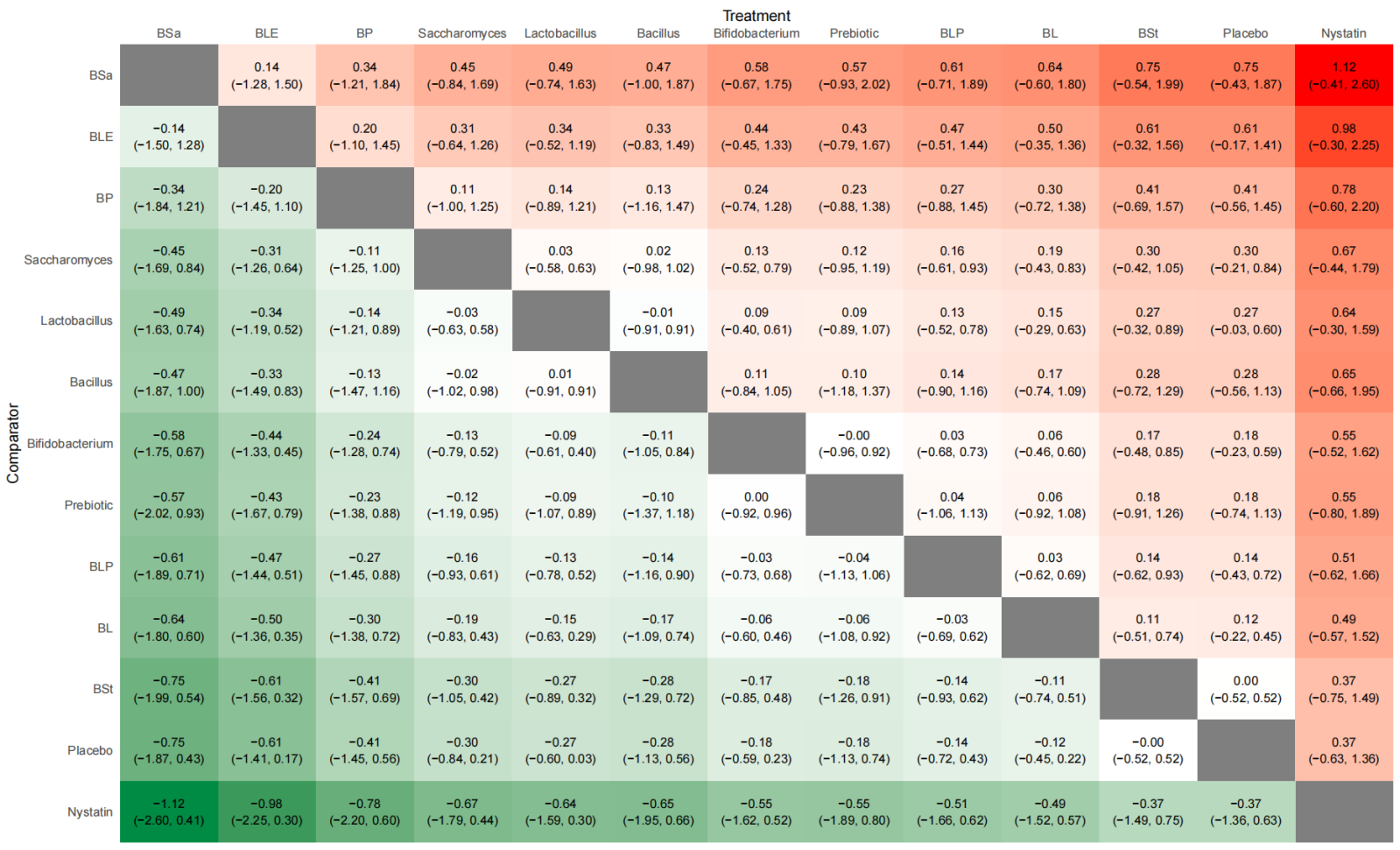
**

**Figure S4: League heat plot of the network estimates.** The table generated by the nma.league() function in BUGSnet presents the effectiveness of interventions for primary outcomes. The values in each cell indicate the relative treatment effect (and 95% credible intervals) of the treatment on the top, compared to the treatment on the left, for (a) mortality, (b) incidence of necrotizing enterocolitis (at or beyond Bell Stage II), (c) length of hospital stay, (d) time to reach full feeding, and (e) incidence of culture-confirmed sepsis. The logarithm of RR (with 95% CI) for the column-defining treatment in comparison to the row-defining treatment is displayed in the results. Statistical significance is indicated by a double asterisk.

| **a** | 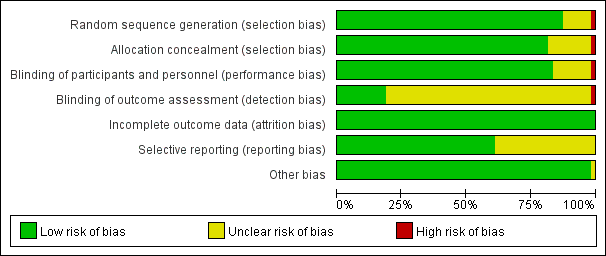  **Figure S5: Risk of bias assessment.** Summary of each risk of bias item as a percentage across all studies (a) and the risk of bias for each included study (b). | **b** | 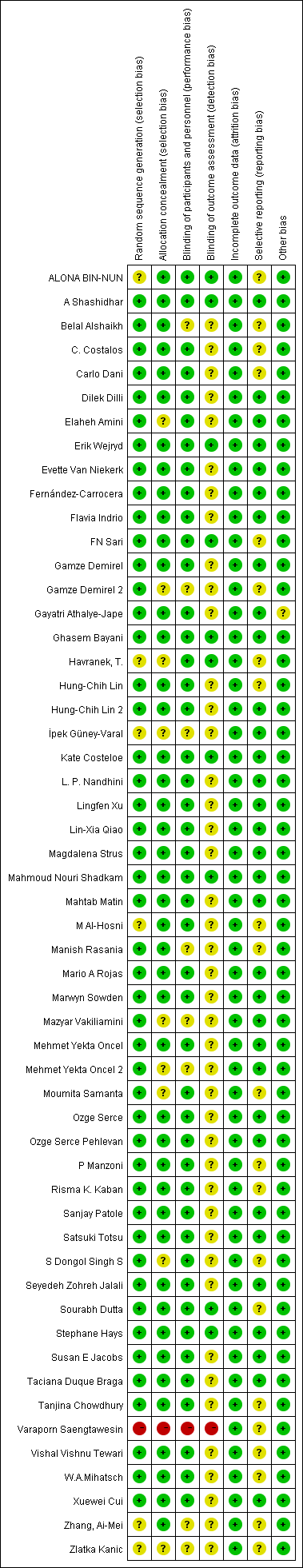 |
| --- | --- | --- | --- |

**
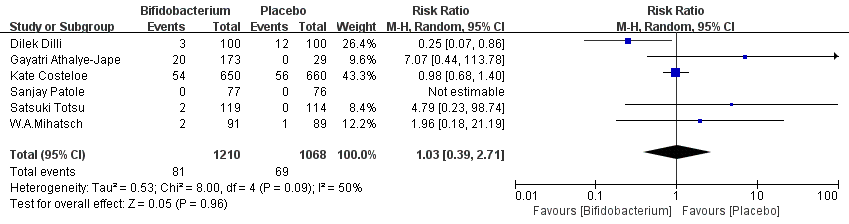
**

**a. Placebo vs Bifidobacterium, outcome: (i) mortality.**

**
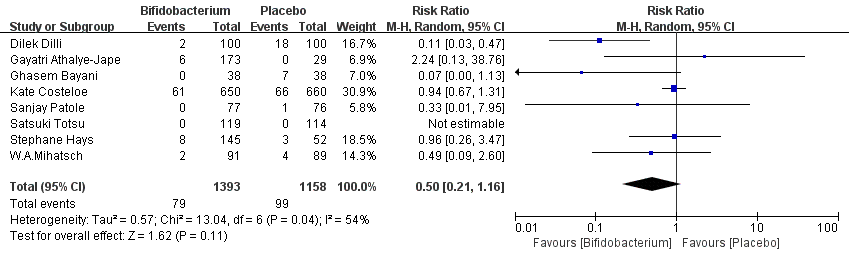
**

**a. Placebo vs Bifidobacterium, outcome: (ii) incidence of NEC (at or beyond Bell Stage II).**

**
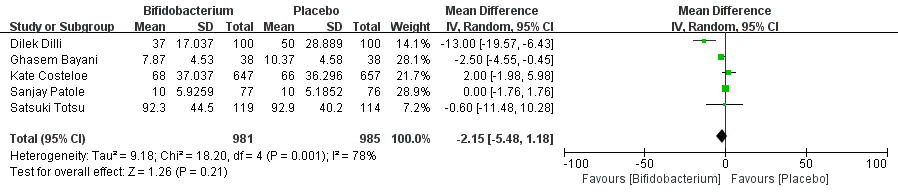
**

**a. Placebo vs Bifidobacterium, outcome: (iii) length of hospital stay.**

**
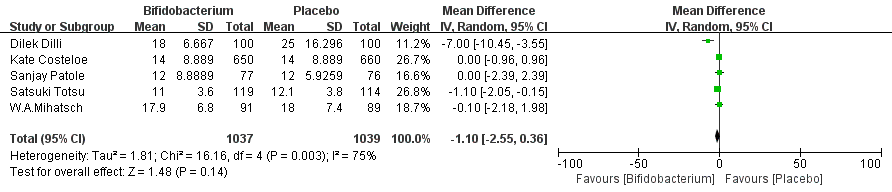
**

**a. Placebo vs Bifidobacterium, outcome: (iv) time to reach full feeding.**

**
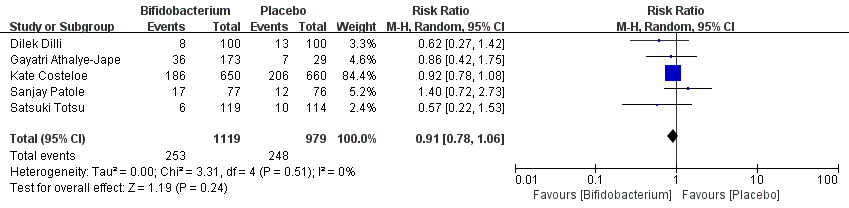
**

**a. Placebo vs Bifidobacterium, outcome: (v) incidence of culture-confirmed sepsis.**

**
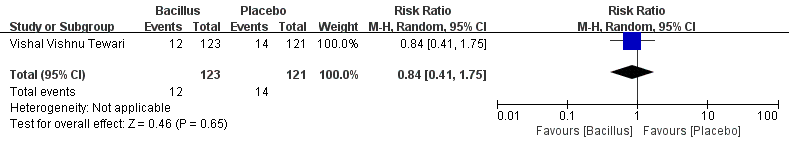
**

**b. Placebo vs Bacillus, outcome: (i) mortality.**

**
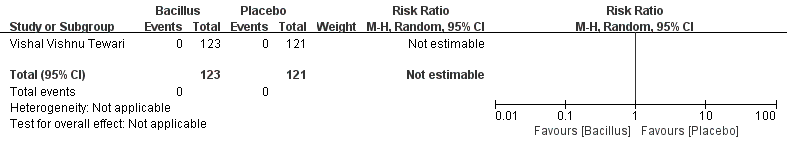
**

**b. Placebo vs Bacillus, outcome: (ii) incidence of NEC (at or beyond Bell Stage II).**

**
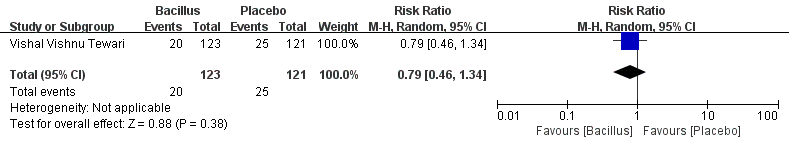
**

**b. Placebo vs Bacillus, outcome: (v) incidence of culture-confirmed sepsis.**

**
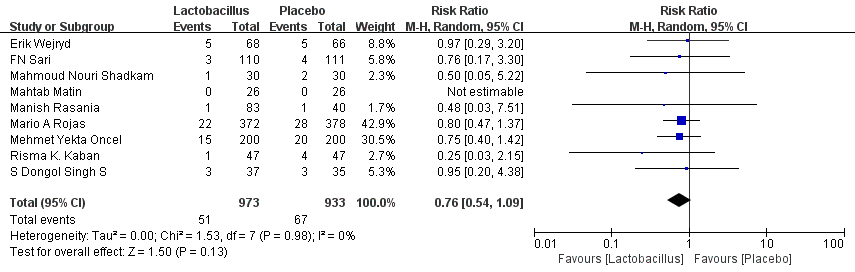
**

**c. Placebo vs Lactobacillus, outcome: (i) mortality.**

**
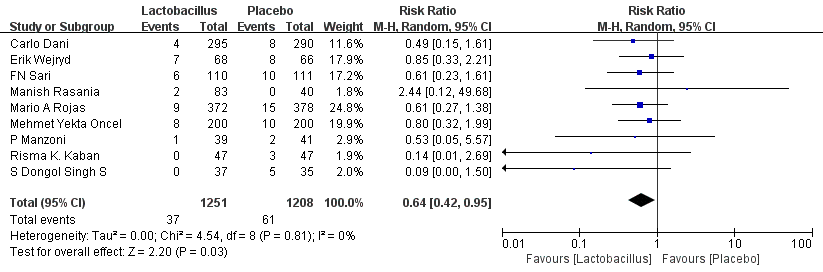
**

**c. Placebo vs Lactobacillus, outcome: (ii) incidence of NEC (at or beyond Bell Stage II).**

**
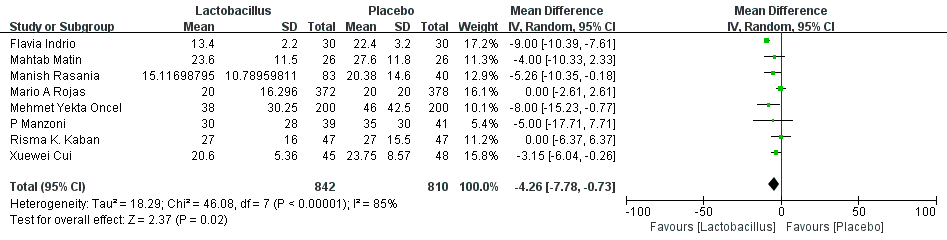
**

**c. Placebo vs Lactobacillus, outcome: (iii) length of hospital stay.**

**
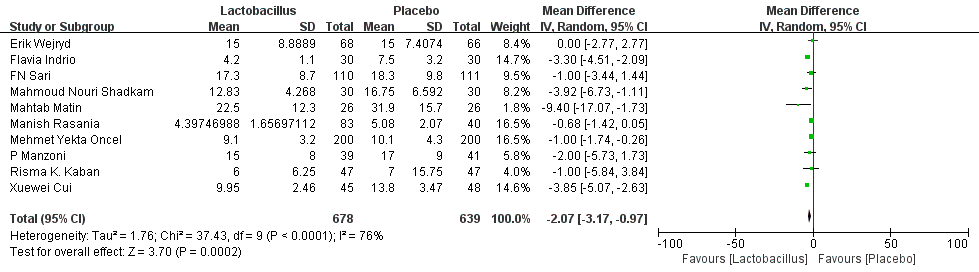
**

**c. Placebo vs Lactobacillus, outcome: (iv) time to reach full feeding.**

**
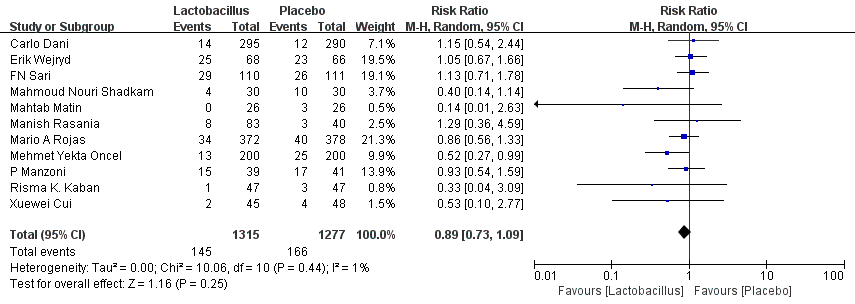
**

**c. Placebo vs Lactobacillus, outcome: (v) incidence of culture-confirmed sepsis.**

**
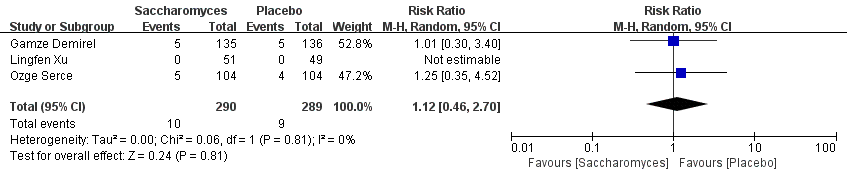
**

**d. Placebo vs Saccharomyces, outcome: (i) mortality.**

**
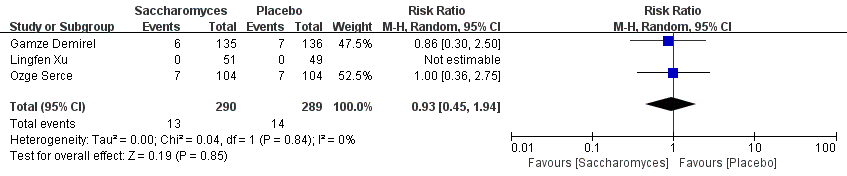
**

**d. Placebo vs Saccharomyces, outcome: (ii) incidence of NEC (at or beyond Bell Stage II).**

**
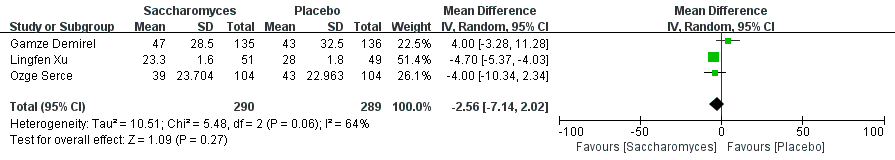
**

**d. Placebo vs Saccharomyces, outcome: (iii) length of hospital stay.**

**
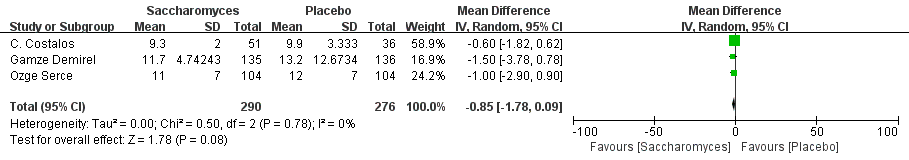
**

**d. Placebo vs Saccharomyces, outcome: (iv) time to reach full feeding.**

**
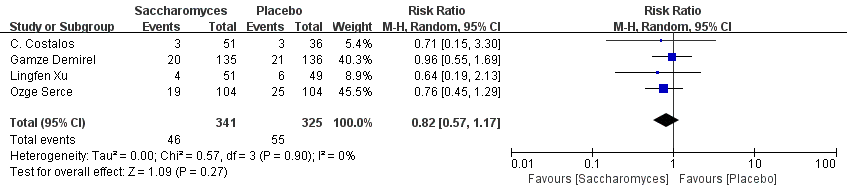
**

**d. Placebo vs Saccharomyces, outcome: (v) incidence of culture-confirmed sepsis.**

**
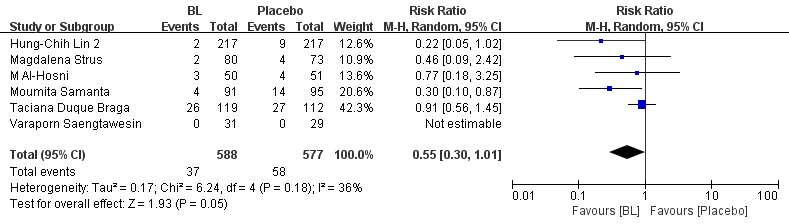
**

**e. Placebo vs BL, outcome: (i) mortality.**

**
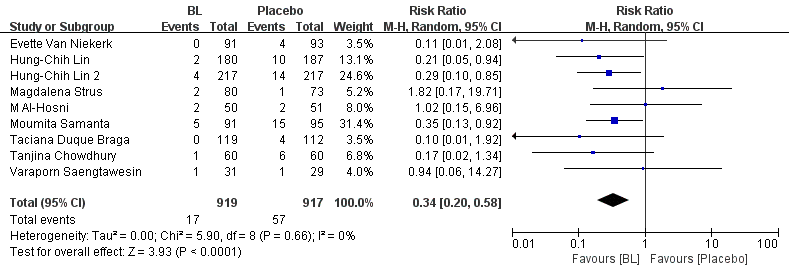
**

**e. Placebo vs BL, outcome: (ii) incidence of NEC (at or beyond Bell Stage II).**

**
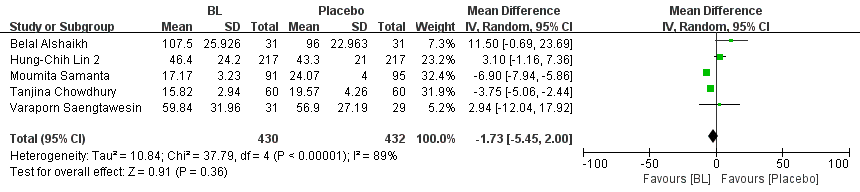
**

**e. Placebo vs BL, outcome: (iii) length of hospital stay.**

**
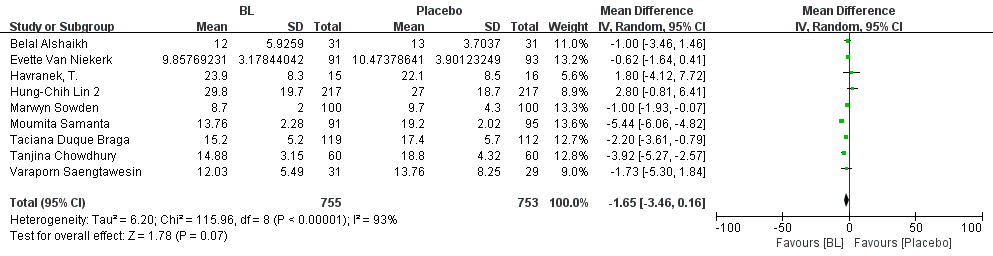
**

**e. Placebo vs BL, outcome: (iv) time to reach full feeding.**

**
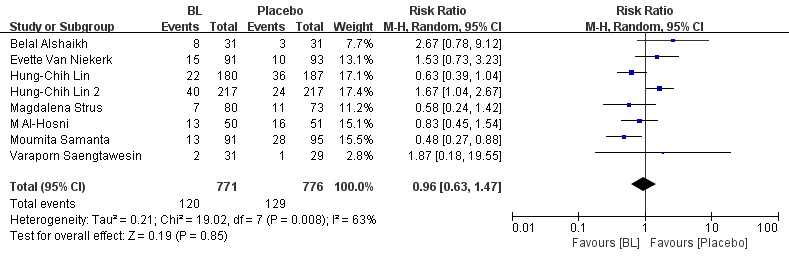
**

**e. Placebo vs BL, outcome: (v) incidence of culture-confirmed sepsis.**

**
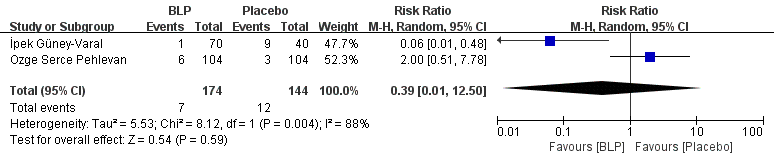
**

**f. Placebo vs BLP, outcome: (i) mortality.**

**
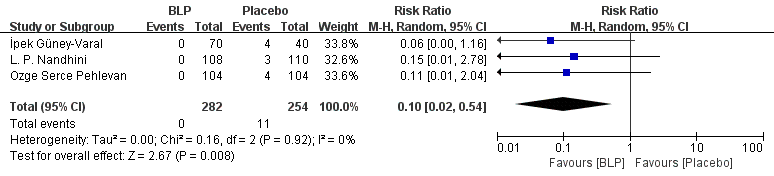
**

**f. Placebo vs BLP, outcome: (ii) incidence of NEC (at or beyond Bell Stage II).**

**
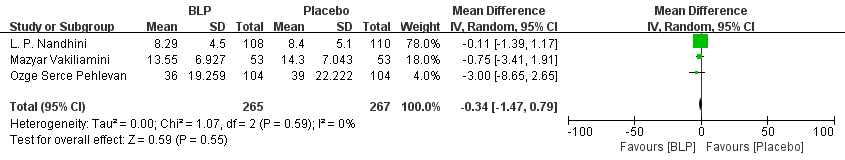
**

**f. Placebo vs BLP, outcome: (iii) length of hospital stay.**

**
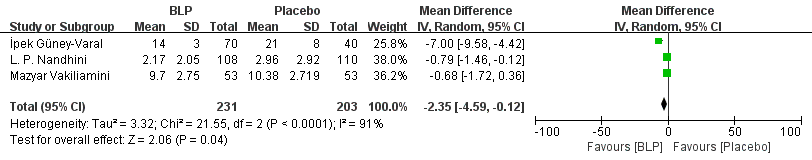
**

**f. Placebo vs BLP, outcome: (iv) time to reach full feeding.**

**
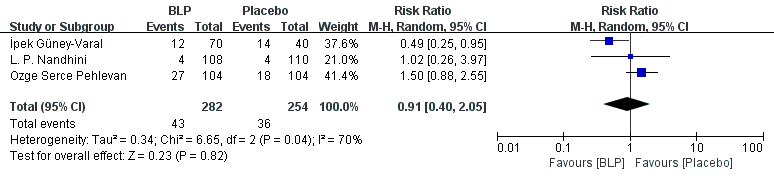
**

**f. Placebo vs BLP, outcome: (v) incidence of culture-confirmed sepsis.**

**
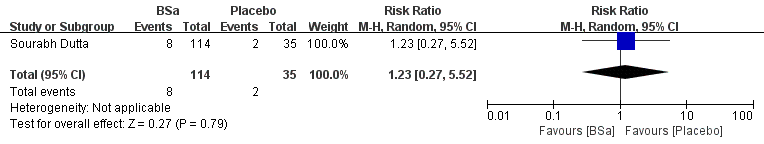
**

**g. Placebo vs BSa, outcome: (i) mortality.**

**
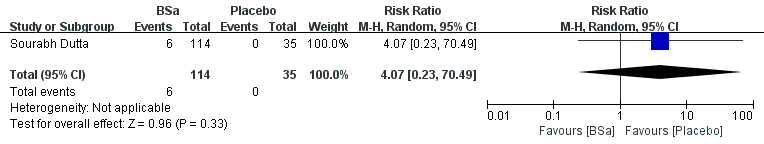
**

**g. Placebo vs BSa, outcome: (ii) incidence of NEC (at or beyond Bell Stage II).**

**
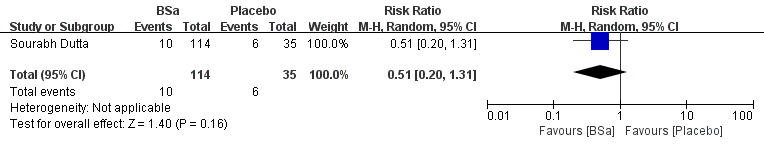
**

**g. Placebo vs BSa, outcome: (v) incidence of culture-confirmed sepsis.**

**
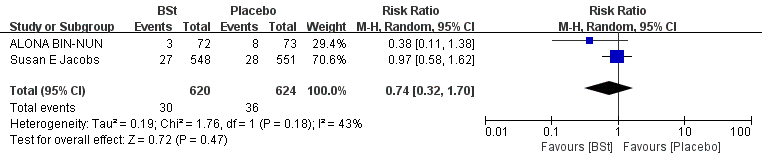
**

**h. Placebo vs BSt, outcome: (i) mortality.**

**
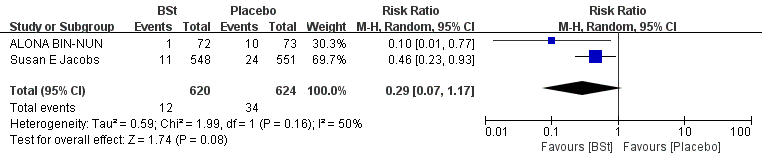
**

**h. Placebo vs BSt, outcome: (ii) incidence of NEC (at or beyond Bell Stage II).**

**
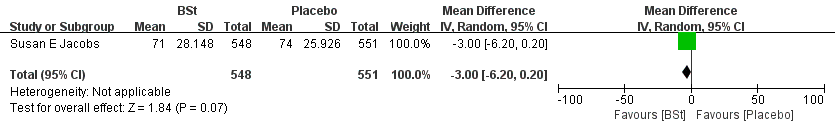
**

**h. Placebo vs BSt, outcome: (iii) length of hospital stay.**

**
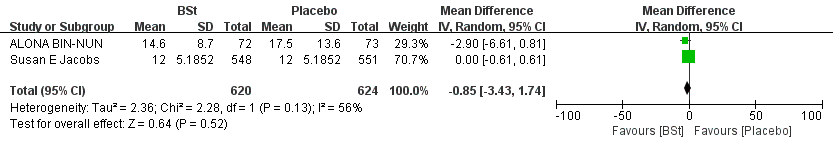
**

**h. Placebo vs BSt, outcome: (iv) time to reach full feeding.**

**
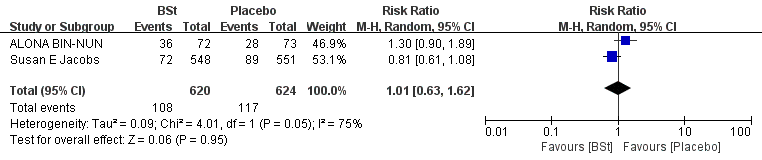
**

**h. Placebo vs BSt, outcome: (v) incidence of culture-confirmed sepsis.**

**
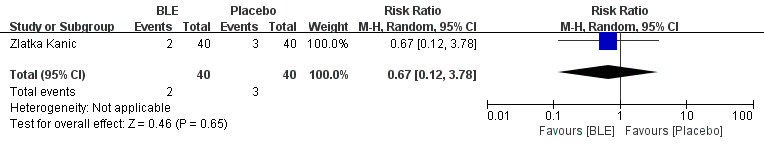
**

**i. Placebo vs BLE, outcome: (i) mortality.**

**
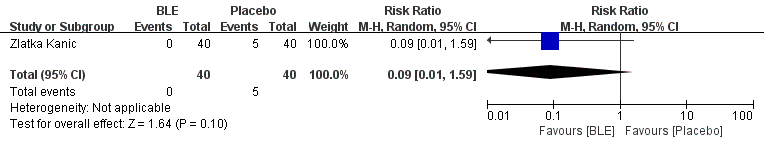
**

**i. Placebo vs BLE, outcome: (ii) incidence of NEC (at or beyond Bell Stage II).**

**
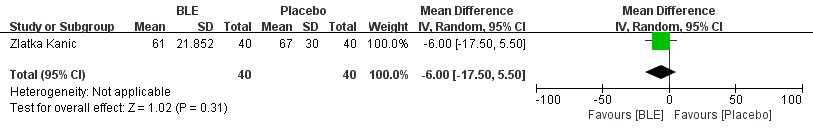
**

**i. Placebo vs BLE, outcome: (iii) length of hospital stay.**

**
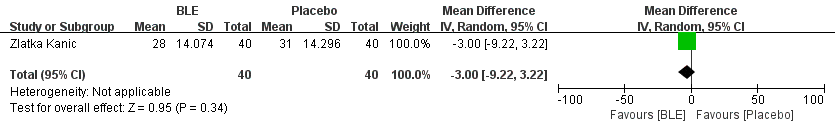
**

**i. Placebo vs BLE, outcome: (iv) time to reach full feeding.**

**
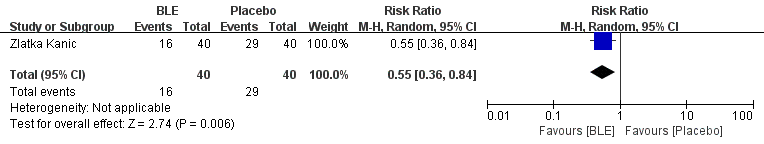
**

**i. Placebo vs BLE, outcome: (v) incidence of culture-confirmed sepsis.**

**
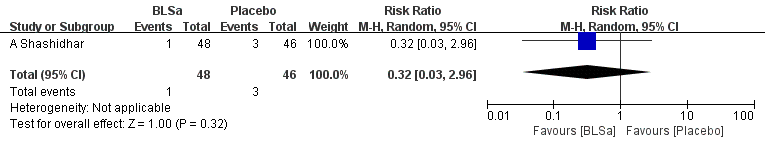
**

**j. Placebo vs BLSa, outcome: (i) mortality.**

**
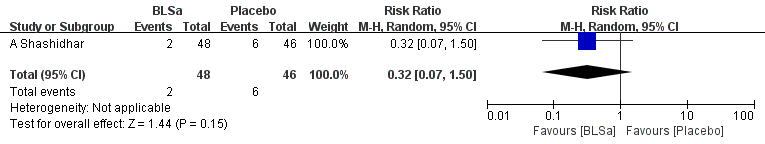
**

**j. Placebo vs BLSa, outcome: (ii) incidence of NEC (at or beyond Bell Stage II).**

**
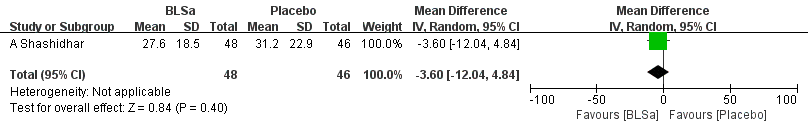
**

**j. Placebo vs BLSa, outcome: (iii) length of hospital stay.**

**
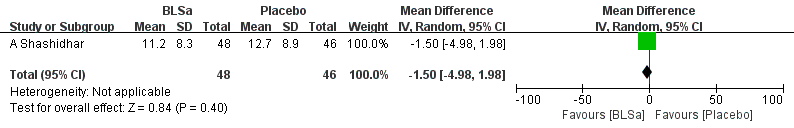
**

**j. Placebo vs BLSa, outcome: (iv) time to reach full feeding.**

**
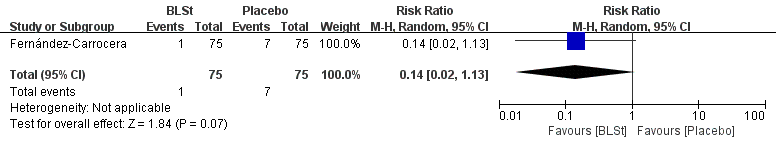
**

**k. Placebo vs BLSt, outcome: (i) mortality.**

**
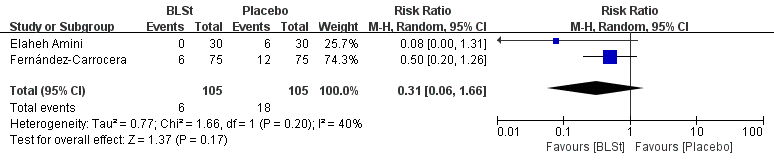
**

**k. Placebo vs BLSt, outcome: (ii) incidence of NEC (at or beyond Bell Stage II).**

**
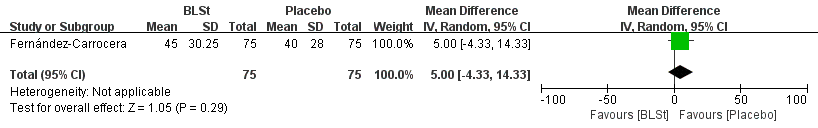
**

**k. Placebo vs BLSt, outcome: (iii) length of hospital stay.**

**
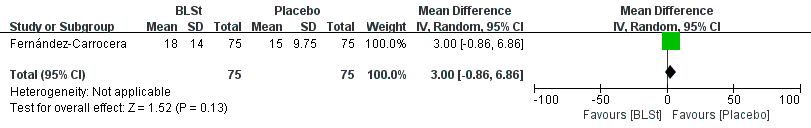
**

**k. Placebo vs BLSt, outcome: (iv) time to reach full feeding.**

**
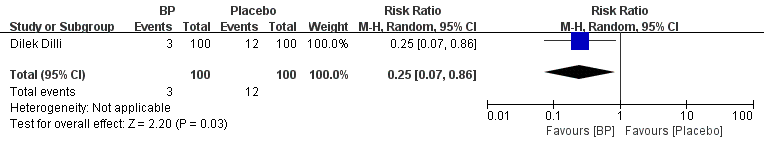
**

**l. Placebo vs BP, outcome: (i) mortality.**

**
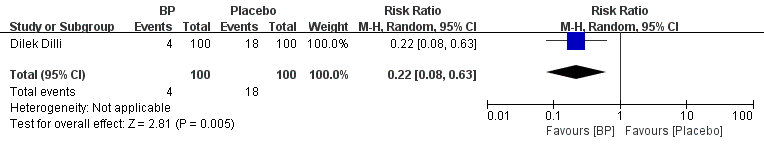
**

**l. Placebo vs BP, outcome: (ii) incidence of NEC (at or beyond Bell Stage II).**

**
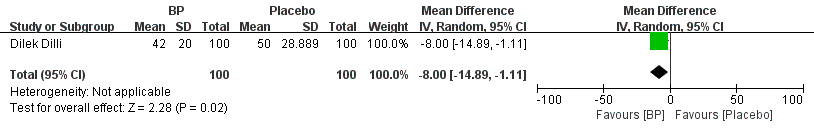
**

**l. Placebo vs BP, outcome: (iii) length of hospital stay.**

**
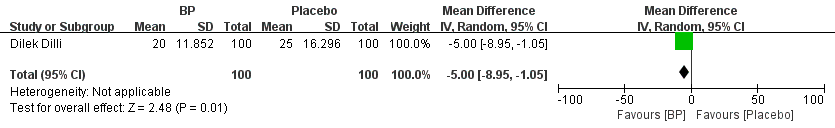
**

**l. Placebo vs BP, outcome: (iv) time to reach full feeding.**

**
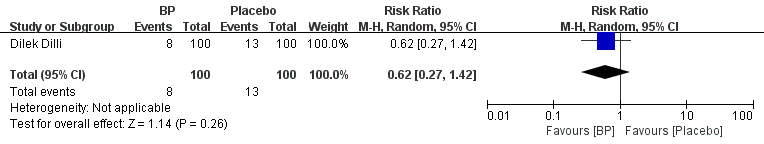
**

**l. Placebo vs BP, outcome: (v) incidence of culture-confirmed sepsis.**

**
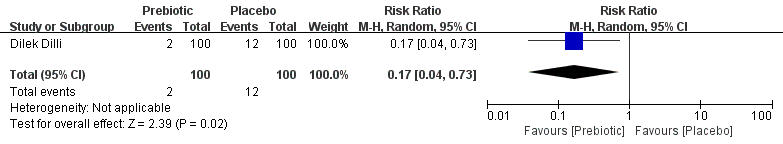
**

**m. Placebo vs Prebiotic, outcome: (i) mortality.**

**
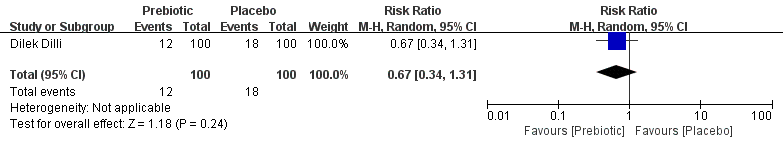
**

**m. Placebo vs Prebiotic, outcome: (ii) incidence of NEC (at or beyond Bell Stage II).**

**
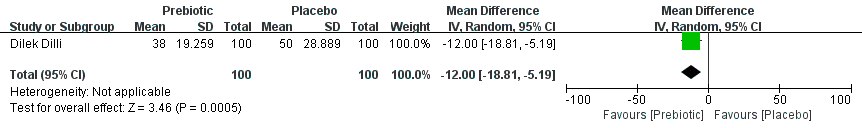
**

**m. Placebo vs Prebiotic, outcome: (iii) length of hospital stay.**

**
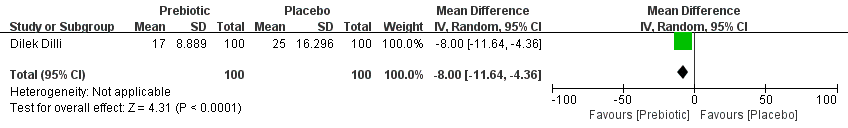
**

**m. Placebo vs Prebiotic, outcome: (iv) time to reach full feeding.**

**
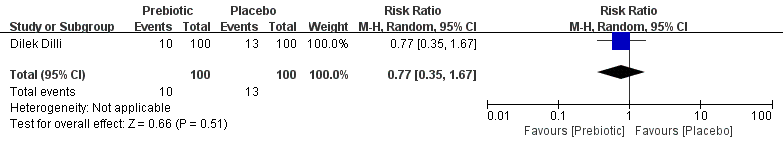
**

**m. Placebo vs Prebiotic, outcome: (v) incidence of culture-confirmed sepsis.**

**
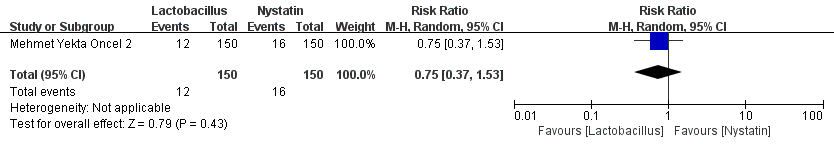
**

**n. Nystatin vs Lactobacillus, outcome: (i) mortality.**

**
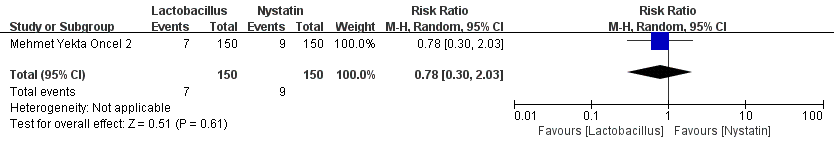
**

**n. Nystatin vs Lactobacillus, outcome: (ii) incidence of NEC (at or beyond Bell Stage II).**

**
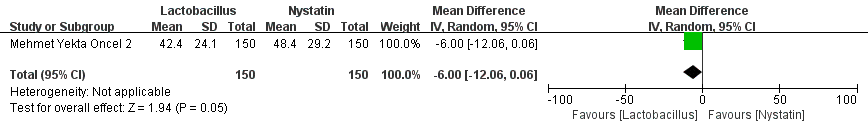
**

**n. Nystatin vs Lactobacillus, outcome: (iii) length of hospital stay.**

**
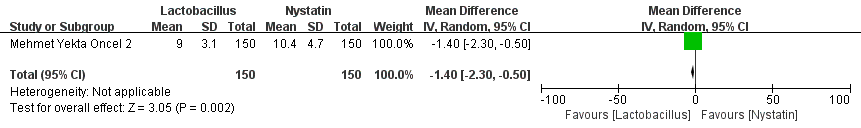
**

**n. Nystatin vs Lactobacillus, outcome: (iv) time to reach full feeding.**

**
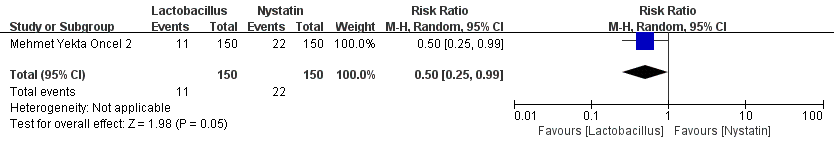
**

**n. Nystatin vs Lactobacillus, outcome: (v) incidence of culture-confirmed sepsis.**

**
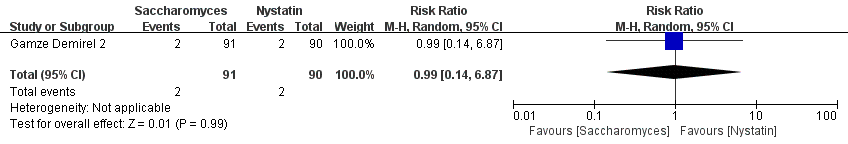
**

**o. Nystatin vs Saccharomyces, outcome: (i) mortality.**

**
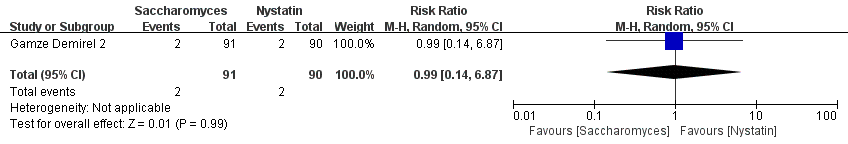
**

**o. Nystatin vs Saccharomyces, outcome: (ii) incidence of NEC (at or beyond Bell Stage II).**

**
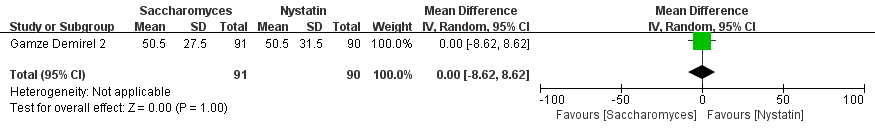
**

**o. Nystatin vs Saccharomyces, outcome: (iii) length of hospital stay**

**Figure S6: Forest plot of two direct comparisons.** Fifteen intervention types (a-o) were compared based on five key outcome indicators: (i) mortality, (ii) incidence of necrotizing enterocolitis (at or beyond Bell Stage II), (iii) length of hospital stay, (iv) time to reach full feeding, and (v) incidence of culture-confirmed sepsis.
