# Supplementary material for: Effect of probiotics on necrotizing enterocolitis in preterm infants: a network meta-analysis of randomized controlled trials
Source: BMC Pediatr. 2025 Mar 27;25:237. doi: 10.1186/s12887-025-05469-z (PMC11948853; doi:10.1186/s12887-025-05469-z)
Supplement: Supplementary file 2 — Supplementary Material 2 [file 12887_2025_5469_MOESM2_ESM.docx]

Supplement tables

# Effect of Probiotics on Necrotizing Enterocolitis in Preterm Infants: A Network Meta-Analysis of Randomized Controlled Trials

Yu Dai^1,#^, Qinlei Yu^1,#^, Fan Zhang^1^, Ke Ma^1^, Xiangyun Yan^1^, Wenjuan Chen^1^, Xiaohui Chen^1,*^, Shushu Li^1,*^, Shuping Han^1,*^

^1^Department of Pediatrics, Women’s Hospital of Nanjing Medical University, Nanjing Women and Children’s Healthcare Hospital, Nanjing, China.

^#^ These authors contributed equally to this work.

**^*^** Corresponding author: shupinghan@njmu.edu.cn (S.P. Han), lishushu@njmu.edu.cn (S.S. Li), chenxiaohui@njmu.edu.cn (X.H. Chen)

**Table S1: Search strategies**

**Table S2: Characteristics of the included patients**

**Table S3: Classification of the included interventions**

**Table S4: Primary outcomes**

**Table S1: Search strategies**

| **Database** | **Query** |
| --- | --- |
| PubMed | ((preterm infant OR pre-term infant) OR (preterm infants OR pre-term infants) OR (preterm neonate OR pre-term neonate) OR (preterm neonates OR pre-term neonates) OR (preterm newborn OR pre-term newborn) OR (preterm newborns OR pre-term newborns) OR (premature infant OR premature infants) OR (premature neonate OR premature neonates) OR (premature newborn OR premature newborns) OR infant, extremely premature [MH] OR premature birth [MH] OR infant, low birth weight [MH] OR infant, very low birth weight [MH])) AND ((feeding difficulties) OR (feeding intolerance) OR (feed intolerance) OR (necrotizing enterocolitis)) AND (probiotic* OR synbiotic*) |
| The Cochrane Library | 296 Trials matching 'preterm infant' OR 'pre-term infant' OR 'preterm infants' OR 'pre-term infants' OR 'preterm neonate' OR 'pre-term neonate' OR 'preterm neonates' OR 'pre-term neonates' OR 'preterm newborn' OR 'pre-term newborn' OR 'preterm newborns' OR 'pre-term newborns' OR 'premature infant' OR 'premature infants' OR 'premature neonate' OR 'premature neonates' OR 'premature newborn' OR 'premature newborns' OR 'extremely premature' OR 'premature birth' OR 'low birth weight' OR 'very low birth weight' in All Text AND 'feeding difficulties' OR 'feeding intolerance' OR 'feed intolerance' OR 'necrotizing enterocolitis' in All Text AND probiotic* OR synbiotic* in All Text - (Word variations have been searched) |
| Web of Science | ((TS=('preterm infant' OR 'pre-term infant' OR 'preterm infants' OR 'pre-term infants' OR 'preterm neonate' OR 'pre-term neonate' OR 'preterm neonates' OR 'pre-term neonates' OR 'preterm newborn' OR 'pre-term newborn' OR 'preterm newborns' OR 'pre-term newborns' OR 'premature infant' OR 'premature infants' OR 'premature neonate' OR 'premature neonates' OR 'premature newborn' OR 'premature newborns' OR 'extremely premature' OR 'premature birth' OR 'low birth weight' OR 'very low birth weight')) AND TS=('feeding difficulties' OR 'feeding intolerance' OR 'feed intolerance' OR 'necrotizing enterocolitis')) AND TS=(probiotic* OR synbiotic*) |
| Embase | ('preterm infant'/exp OR 'preterm infant' OR 'pre-term infant'/exp OR 'pre-term infant' OR 'preterm infants' OR 'pre-term infants' OR 'preterm neonate'/exp OR 'preterm neonate' OR 'pre-term neonate'/exp OR 'pre-term neonate' OR 'preterm neonates' OR 'pre-term neonates' OR 'preterm newborn'/exp OR 'preterm newborn' OR 'pre-term newborn'/exp OR 'pre-term newborn' OR 'preterm newborns' OR 'pre-term newborns' OR 'premature infant'/exp OR 'premature infant' OR 'premature infants' OR 'premature neonate'/exp OR 'premature neonate' OR 'premature neonates' OR 'premature newborn'/exp OR 'premature newborn' OR 'premature newborns' OR 'extremely premature' OR 'premature birth'/exp OR 'premature birth' OR 'low birth weight'/exp OR 'low birth weight' OR 'very low birth weight'/exp OR 'very low birth weight') AND ('feeding difficulties' OR 'feeding intolerance'/exp OR 'feeding intolerance' OR 'feed intolerance' OR 'necrotizing enterocolitis'/exp OR 'necrotizing enterocolitis') AND (probiotic* OR synbiotic*) |
| OVID | ((preterm infant OR pre-term infant) OR (preterm infants OR pre-term infants) OR (preterm neonate OR pre-term neonate) OR (preterm neonates OR pre-term neonates) OR (preterm newborn OR pre-term newborn) OR (preterm newborns OR pre-term newborns) OR (premature infant OR premature infants) OR (premature neonate OR premature neonates) OR (premature newborn OR premature newborns) OR infant, extremely premature [MH] OR premature birth [MH] OR infant, low birth weight [MH] OR infant, very low birth weight [MH])) AND ((feeding difficulties) OR (feeding intolerance) OR (feed intolerance) OR (necrotizing enterocolitis)) AND (probiotic* OR synbiotic*) |

**Table S2: Characteristics of the included patients**

|  | **Male [n(%)]** | | **Birth weight [g, Mean±SD]** | | **Gestational age [week, Mean±SD]** | | **Caesarean section [n(%)]** | | **Multiple births [n(%)]** | | |
| --- | --- | --- | --- | --- | --- | --- | --- | --- | --- | --- | --- |
| **Study** | **Control** | **Intervention** | **Control** | **Intervention** | **Control** | **Intervention** | **Control** | **Intervention** | **Control** | **Intervention** |  |
| Taciana D 2011 | 55(49.1) | 58(48.7) | 1151.4±224.9 | 1194.7±206.3 | 29.2±2.6 | 29.5±2.5 | 55(49.1) | 64(53.8) | 25(22.3) | 19(15.9) |  |
| Lingfen X 2016 | 24(49.0) | 27(52.9) | 1957±51 | 1947±54 | 33±1.04 | 33±0.72 |  |  |  |  |  |
| ALONA 2005 | 37(50.7) | 44(61.1) | 1111±278 | 1152±262 | 29.3±4.3 | 29.8±2.6 | 57(78.1) | 56(77.8) |  |  |  |
| Dilek D 2015 | 58(58.0) | 53(53.0) | 1147±271 | 1236±212 | 28.2±2.2 | 28.8±1.9 | 37(37.0) | 35(35.0) | 9(9.0) | 13(13.0) |  |
|  |  | 52(52.0) |  | 1229±246 |  | 29.0±1.7 |  | 37(37.0) |  | 11(11.0) |  |
|  |  | 57(57.0) |  | 1205±240 |  | 28.8±1.9 |  | 29(29.0) |  | 8(8.0) |  |
| Kate C 2016 | 370(56.1) | 374(57.5) | 1043±317 | 1039±312 | 28.0(26.1-29.6) | 28.0(26.1-29.4) | 349(53.0) | 341(52.5) | 201(30.4) | 193(29.5) |  |
| Stephane H 2015 | 35(67.3) | 66(45.8) | 1170(1055-1370) | 1170(1000-1320) | 29.4(27.9-30.6) | 29.0(28.1-30.1) | 39(75.0) | 115(79.3) |  |  |  |
| Costalos 2003 | 23(63.9) | 24(47.1) | 1644[348.7%] | 1651[470%] | 31.8[2.7%] | 31.1[2.5%] | 14(38.9) | 25(49.0) |  |  |  |
| Manzoni 2006 | 21(51.2) | 20(51.3) | 1174±340 | 1212±290 | 29.3±4 | 29.6±5 | 27(65.9) | 26(66.7) |  |  |  |
| Shashidhar 2017 | 20(38.5) | 27(51.9) | 1190±208 | 1256±185 | 31.2±2.1 | 31.2±2.1 | 38(73.1) | 27(51.9) |  |  |  |
| Elaheh A 2017 |  |  | 1162.33 | 1144.23 | 29.63 | 29.47 |  |  |  |  |  |
| Carlo D 2002 | 151(52.1) | 135(45.8) | 1345±384 | 1325±361 | 30.7±2.3 | 30.8±2.4 | 239(82.4) | 225(76.3) |  |  |  |
| W.A.M 2010 | 47(52.2) | 55(59.1) | 871±287 | 856±251 | 26.7±1.7 | 26.6±1.8 |  |  | 29(32.2) | 29(31.2) |  |
| M.A.H 2012 | 28(54.9) | 22(44.0) | 779±126 | 778±138 | 25.7±1.4 | 25.7±1.4 | 30(58.8) | 22(44.0) | 10(19.6) | 8(16.0) |  |
| G.A.J 2022 | 16(55.2) | 48(55.8) | 810(685-970) | 828(679-971) | 26.1(25.2–26.9) | 26.2(24.4–27.2) | 13(44.8) | 52(60.5) |  |  |  |
|  |  | 46(52.9) |  | 870(700-1050) |  | 26.3(24.7–27.1) |  | 47(54.0) |  |  |  |
| O.S.P 2020 | 61(58.7) | 52(50.0) | 1151±269 | 1197±235 | 28±2.2 | 29±1.9 | 94(90.4) | 91(87.5) |  |  |  |
| Chowdhury 2016 | 36(72.0) | 33(63.5) | 1338.0±97.71 | 1310.6±110.41 | 31.68±0.84 | 31.38±0.93 |  |  |  |  |  |
| Gamze D 2013 | 66(48.5) | 69(51.1) | 1131±284 | 1164±261 | 29.2±2.5 | 29.4±2.3 | 113(83.0) | 105(77.7) |  |  |  |
| S Dongol Singh S 2017 | 16(45.7) | 16(43.2) |  |  | 32.6±2.2 | 32.6±2.2 | 7(20.0) | 8(21.6) |  |  |  |
| Fernández 2013 |  |  | 1170(540,1492) | 1090(580,1495) | 31[27,36] | 31.2[26,35.4] |  |  |  |  |  |
| Moumita S 2009 |  |  | 1210±143 | 1172±143 | 30.14±1.59 | 30.12±1.63 | 47(49.5) | 42(46.2) | 19(20.0) | 14(15.1) |  |
| E.V.N 2015 | 24(42.9) | 29(53.7) | 1001–1250g: 23(41%) | 29(53%) | 24–28weeks: 31(56%) | 23(43%) | HIV-free mothers | 81(73.6) |  |  |  |
|  |  | 19(51.4) |  | 21(57%) |  | 16(46%) |  |  |  |  |  |
|  |  | 14(37.8) |  | 19(51%) |  | 18(53%) | HIV mothers | 58(78.4) |  |  |  |
| V.V.T 2015 | 32(51.6) | 29(46.8) | 1260±282 (32%) | 1280±208 (27%) | 31.57±0.29 (69%) | 31.29±0.29 (68%) | 37(60.0) | 36(58.1) | 16(25.8) | 17(27.4) |  |
|  |  | 31(52.5) |  | 713±36 (10%) |  | 27.43±0.43 (45%) |  | 35(59.3) |  | 20(33.9) |  |
|  |  | 32(52.5) |  | 715±29 (9.8%) |  | 27.0±0.43 (46%) |  | 40(65.6) |  | 17(27.9) |  |
| Havranek 2013 | 7(43.8) | 8(53.3) | 789±129 | 856±105 | 25.9±1.5 | 25.9±1.3 | 10(62.5) | 7(46.7) |  |  |  |
| M Strus 2018 | 50(56.8) | 42(47.2) | 1350.11±292.18 | 1281.24±281.18 | 29.67±2.32 | 29.73±2.26 | 77(87.5) | 75(84.3) | 28(31.8) | 25(28.1) |  |
| Susan E 2013 | 300(54.4) | 272(49.6) | 1048±260 | 1063±259 | 27.8±2.0 | 27.9±2.0 | 377(68.4) | 359(65.5) | 193(35.0) | 197(35.9) |  |
| Risma K 2019 | 17(36.2) | 28(59.6) | 1605(1060,1800) | 1520(1035,1800) | 33(28,34) | 33(28,34) | 40(85.1) | 39(83.0) |  |  |  |
| H.C.L 2005 | 100(53.5) | 84(46.7) | 1071±243 | 1104±242 | 28.2±2.5 | 28.5±2.5 | 100(53.5) | 104(57.8) | 33(17.6) | 34(18.9) |  |
| H.C.L 2008 | 115(53) | 122(56.2) | 1077.3±214.4 | 1028.9±246.0 |  |  | 136(63.3) | 149(69.6) |  |  |  |
| Erik W 2018 | 42(63.6) | 32(47.1) | 740±148 | 731±129 | 25.5±1.3 | 25.5±1.2 | 37(56.1) | 50(73.5) | 19(28.8) | 28(41.2) |  |
| Mazyar V 2020 | 24(45.3) | 33(62.3) | 1862.26±426.148 | 1789.62±441.196 | 33.08±2.046 | 32.32±2.471 | 25(47.2) | 27(51.0) |  |  |  |
| Belal A 2022 | 21(67.7) | 16(51.6) | 751±132 | 763±209 | 25.6±1.3 | 25.8±1.5 | 25(80.7） | 23(74.2) | 8(25.8) | 10(32.3) |  |
| L.P.N 2015 |  |  | 1444±217 | 1430±209 | 31.4±1.4 | 31.6±1.4 | 11(10.0) | 9(8.3) | 44(40.0) | 38(34.5) |  |
| Satsuki T 2014 | 71(54.6) | 87(56.9) | 998±281 | 1016±289 | 28.5±3.3 | 28.6±2.9 | 103(79.2) | 91(59.5) | 27(20.8) | 29(19.0) |  |
| Flavia I 2017 | 16(53.3) | 15(50.0) | 1406.6±536.4 | 1471.5±455.1 | 30.1±1.2 | 30.2±1.2 | 25(83.3) | 26(86.7) |  |  |  |
| Marwyn S 2022 | 37(37.0) | 47(47.0) | 1150±230 | 1174±226 | 29±1.98 | 30±1.93 | 73(73.0) | 73(73.0) | 14(14.0) | 21(21.0) |  |
| M.Y.O 2014 | 98(49.0) | 108(54.0) | 1048±298 | 1071±274 | 27.9±2.5 | 28.2±2.4 | 152(76.0) | 163(81.5) |  |  |  |
| M.Y.O 2015 | 75(50.0) | 82(54.7) | 1015±320 | 1058±270 | 27.7±2.5 | 28.1±2.4 | 110(73.3) | 120(80.0) | 34(22.7) | 38(25.3) |  |
| İpek Güney V 2017 | 19(47.5) | 45(64.3) | 1228±249 | 1728.5±257 | 29.3±1.7 | 29.7±1.9 | 31(77.5) | 58(82.0) | 6(15.0) | 21(30.0) |  |
| M.A.R 2012 | 185(48.9) | 186(50.0) | 1516(1129-1750) | 1530(1253-1750) | 32(29-33) | 32(30-33) | 310(82.0) | 312(83.9) |  |  |  |
| Sanjay P 2014 | 41(53.9) | 45(58.4) | 1025(810-1260) | 1090(755-1280) | 28(26-29) | 29(26-30) | 49(64.5) | 58(75.3) |  |  |  |
| Sourabh D 2015 | 23(65.7) | 25(65.8) | 1252.27±309.31 | 1286.08±264.76 | 30.82±1.72 | 30.64±1.64 | 14(40.0) | 8(21.1) |  |  |  |
|  |  | 23(60.5) |  | 1335.97±284.45 |  | 31.08±1.88 |  | 14(36.8) |  |  |  |
|  |  | 20(52.6) |  | 1413.32±296.56 |  | 30.89±1.96 |  | 13(34.2) |  |  |  |
| Ghasem B 2021 |  |  | 1942.63±581.29 | 1958.68±484.31 | 32.84±1.83 | 32.81±1.81 | 29(76.3) | 30(78.9) |  |  |  |
| Mahtab M 2022 | 13(50.0) | 9(34.6) | 1362±143 | 1386±109 | 30.8±2.3 | 32.0±2.4 |  |  |  |  |  |
|  |  | 13(50.0) |  | 1396±139 |  | 31.7±2.2 |  |  |  |  |  |
| Gamze D 2013 | 50(55.6) | 45(49.5) | 1057±290 | 1135±253 | 28.4±2.6 | 29.0±2.7 | 74(82.2) | 72(79.1) |  |  |  |
| Zlatka K 2015 | 27(67.5) | 22(55.0) | 1024.3±249.9 | 1104.1±233.2 | 29.0(26.2-30.0) | 28.0(27.0-30.0) |  |  | 8(20.0) | 15(37.5) |  |
| Xuewei C 2019 | 20(41.7) | 25(55.6) | 1714±127.11 | 1682±109.03 | 32.56±1.41 | 32.85±1.39 |  |  |  |  |  |
| Varaporn S 2014 | 11(37.9) | 19(61.3) | 1,207.72±199.35 | 1,250.1±179.26 | 30.59±1.76 | 31.0±1.82 | 18(62.0) | 21(67.7) |  |  |  |
| FN Sari 2011 | 62(55.9) | 60(54.5) | 1278±282 | 1231±262 | 29.7±2.4 | 29.5±2.4 | 84(75.7) | 74(67.3) | 42(37.8) | 37(33.6) |  |
| Ozge S 2013 | 56(53.8) | 51(49) | 1162±216 | 1126±232 | 28.7±2.1 | 28.8±2.2 | 92(88.5) | 84(80.8) |  |  |  |
| M.N.S 2015 |  |  | 1418.67±328.47 | 1396.33±234.55 | 30.97±1.94 | 30.87±1.90 |  |  |  |  |  |
| Manish Rasania 2023 | 24(60.0) | 25(61.0) | 1435.75±344.02 | 1578.78±302.13 | 31.78±1.75 | 32.20±1.82 | 16(40.0) | 15(36.6) |  |  |  |
|  |  | 18(42.9) |  | 1549.45±286.89 |  | 32.29±1.6 |  | 23(54.8) |  |  |  |

| (continued) | **Apgar 5min** | | **Feeding type*** | | **Antenatal steroid use [n(%)]** | | **Antibiotics use [day/n(%)]** | | **Age at first feeding [day]** | | **Duration of TPN [day/n(%)]** | | |
| --- | --- | --- | --- | --- | --- | --- | --- | --- | --- | --- | --- | --- | --- |
| **Study** | **Control** | **Intervention** | **Control** | **Intervention** | **Control** | **Intervention** | **Control** | **Intervention** | **Control** | **Intervention** | **Control** | **Intervention** |  |
| Taciana D 2011 | 8.1±1.3 | 7.9±1.5 | H | H | 92(82.1) | 96(82.0) | 101(90.2) | 99(83.2) | 2.6±1.1 | 2.7±1.9 | 84(75.0) | 84(70.6) |  |
| Lingfen X 2016 |  |  | F | F |  |  | 9(18.4) | 11(21.6) |  |  |  |  |  |
| ALONA 2005 | 7±2 | 8±1 | M | M |  |  | 14.9±15.0 | 12.5±10.9 | 2.6±2.3 | 2.7±2.3 | 4.8±5.7 | 5.2±6.0 |  |
| Dilek D 2015 | 7(6-8) | 7(6-8) | M | M | 53(53.0) | 57(57.0) | 27(7-42) | 7(7-27) | 2(2-3) | 2(1-3) | 21(12-34) | 16(10-25) |  |
|  |  | 8(7-8) |  | M |  | 62(62.0) |  | 7(7-27) |  | 2(1-3) |  | 14(9-20) |  |
|  |  | 7(7-8) |  | M |  | 47(47.0) |  | 7(7-27) |  | 2(2-3) |  | 18(10-28) |  |
| Kate C 2016 | ≥7: 531(83%) | 524(83%) | M | M | 607(92.0) | 580(88.7) | 11(4,24) | 10(4,23) | 3(2-4) | 3(2-4) |  |  |  |
| Stephane H 2015 |  |  | H | H |  |  | 6(9.6) | 20(9.7) |  |  |  |  |  |
| Costalos 2003 | <7: 12(33%) | 18(35%) | H | H | 31(86.1) | 45(88.2) |  |  |  |  |  |  |  |
| Manzoni 2006 | 7±1 | 6±2 | H | H | 30(72.5) | 27(69.2) | 14±11 | 12±10 | 2±1 | 2±1 | 12±9 | 13±8 |  |
| Shashidhar 2017 | 8±1 | 8±0.8 | H | H | 27(51.9) | 27(51.9) |  |  | 17(9,47.5) | 15(6,51) |  |  |  |
| Elaheh A 2017 | 7.33 | 7.07 |  |  |  |  |  |  |  |  | 31.37 | 32.13 |  |
| Carlo D 2002 |  |  | M | M | 195(67.4) | 215(72.9) | 250(84.7) | 242(83.4) | 3.8±3.6 | 3.4±3.7 | 14.7±18.7 | 12.8±13.9 |  |
| W.A.M 2010 | 7.8±1.4 | 7.9±1.8 | M | M |  |  | 73(82.0) | 73(80.2) | 5.7±4.3 | 5.0±2.7 |  |  |  |
| M.A.H 2012 | median 8 | 7 | M | M | 43(84.3) | 42(84.0) | 13.0±8.5 | 15.3±9.0 |  |  |  |  |  |
| G.A.J 2022 |  |  | H | H |  |  |  | 4(3–5) |  |  |  | 10(8-15) |  |
|  |  |  |  | H |  |  |  | 3(3–4) |  |  |  | 10(8-16) |  |
| O.S.P 2020 | 7(6-8) | 7(6-8) | M | M | 81(77.9) | 85(81.7) |  |  | 2(1-2) | 2(1-2) |  |  |  |
| Chowdhury 2016 |  |  | H | H |  |  |  |  | 3.42 | 3.33 |  |  |  |
| Gamze D 2013 | median 6 | 6 | M | M | 72(52.9) | 74(54.8) | median 10 | 10 |  |  | 8[3.04-14.84] | 8[6.58-10.94] |  |
| S Dongol Singh S 2017 |  |  |  |  | 24(68.6) | 25(67.6) |  |  |  |  |  |  |  |
| Fernández 2013 | ≥7: 73(97.3%) | 70(93.3%) | M | M | 53(70.7) | 50(66.7) | 7(0,31) | 8(0,43) |  |  | 12(0,69) | 13(4,45) |  |
| Moumita S 2009 | >7: 61 | 66 | H | H | 61(64.1) | 58(63.7) |  |  |  |  |  |  |  |
| E.V.N 2015 | >7 45(80%) | 45(83%) | H | H | HIV-free mothers | 72(65.5) | 4.00±2.47 | 3.39±1.60 |  |  |  |  |  |
|  |  | 22(60%) |  | H |  |  |  | 3.60±1.44 |  |  |  |  |  |
|  |  | 29(81%) |  | H | HIV mothers | 54(73.0) |  | 3.99±1.85 |  |  |  |  |  |
| V.V.T 2015 |  |  | H | H | 49(79.0) | 48(77.4) | 51(82.3) | 54(87.1) | Day1:11(17.7) | 12(19.4) |  |  |  |
|  |  |  |  | H |  | 42(71.2) |  | 52(88.1) |  | 0(0) |  |  |  |
|  |  |  |  | H |  | 47(77.0) |  | 51(83.6) |  | 0(0) |  |  |  |
| Havranek 2013 |  |  | H | H | 15(93.8) | 14(93.3) |  |  |  |  |  |  |  |
| M Strus 2018 |  |  | M | M |  |  | 77(87.5) | 73(82.0) |  |  |  |  |  |
| Susan E 2013 | 8(7–9) | 8(7–9) | M | M | 500(90.7) | 502(91.6) | 2(0-8) | 2(0-7) |  |  | 12(8-18) | 12(8-17) |  |
| Risma K 2019 | 9(5,10) | 9(6,10) | M | M |  |  |  |  | 1(0,14) | 2(0,12) | 9(0,69) | 8(0,35) |  |
| H.C.L 2005 | >7: 94 | 98 | H | H | 114(61.0) | 121(67.2) |  |  |  |  | 13.9±5.0 | 14.7±5.7 |  |
| H.C.L 2008 | <6: 50(23.5%) | 61(28.5%) | M | M | 96(44.7) | 107(49.3) |  |  |  |  |  |  |  |
| Erik W 2018 | 6.4±2.5 | 6.2±2.6 | H | H | 65(98.5) | 67(98.5) | 26.0±15.0 | 28.1±14.3 |  |  |  |  |  |
| Mazyar V 2020 |  |  |  |  |  |  |  |  | 3.13±1.21 | 2.98±1.23 |  |  |  |
| Belal A 2022 | 7(5-7) | 6(5-7) | M | M | 30(96.8) | 28(90.3) | 31(97.0) | 28(90.4) |  |  | 17(13-20) | 20(11-34) |  |
| L.P.N 2015 |  |  | H | H | 30(27.3) | 27(24.5) | 7.06±4.7 | 6.11±4.3 |  |  | 1(0.9) | 0(0) |  |
| Satsuki T 2014 | 7(6-9) | 8(7-9) |  |  | 66(50.8) | 101(66.0) |  |  |  |  |  |  |  |
| Flavia I 2017 |  |  |  |  |  |  | 12.5±7.2 | 4.2±4.3 |  |  |  |  |  |
| Marwyn S 2022 |  |  | M | M |  |  |  |  | 3.0±1.0 | 3.1±1.1 |  |  |  |
| M.Y.O 2014 | 8(5,9) | 8(5,9) | M | M | 154(73.0) | 141(66.2) | 9(0,15) | 7(0,14) | 1(1,5) | 1(1,5) | 8(5,45) | 8(5,45) |  |
| M.Y.O 2015 | 8(5,9) | 8(5,9) | M | M | 113(75.3) | 111(74.0) | 10.1±5 | 9.2±5.6 | 1(1,3) | 1(1,3) | 9.9±6.6 | 8.2±4.5 |  |
| İpek Güney V 2017 | median 7 | 8 | M | M | 21(48.8) | 33(43.4) |  |  |  |  | 22±11 | 13±5 |  |
| M.A.R 2012 | 9(8-9) | 9(8-9) | M | M | 277(73.3) | 270(72.6) |  |  |  |  |  |  |  |
| Sanjay P 2014 | <7: 19(25%) | 14(18%) | M | M | 67(83.8) | 68(86.1) | 6(3-11) | 7(5-10) |  |  |  |  |  |
| Sourabh D 2015 | 9(8-9) | 9(8-9) | M | M | 19(54.3) | 24(63.2) |  |  |  |  |  |  |  |
|  |  | 9(8-9) |  | M |  | 26(68.4) |  |  |  |  |  |  |  |
|  |  | 8.5(8-9) |  | M |  | 22(57.9) |  |  |  |  |  |  |  |
| Ghasem B 2021 | 8.34±1.59 | 8.71±1.85 |  |  | 17(44.7) | 22(57.9) |  |  | 37.68±41.13 | 18±18.32 |  |  |  |
| Mahtab M 2022 | <7: 2(8%) | 2(8%) | H | H |  |  |  |  |  |  | 9.3±7.1 | 9.1±10.2 |  |
|  |  | 3(12%) |  | H |  |  |  |  |  |  |  | 6.8±8.0 |  |
| Gamze D 2013 | 6(4-9) | 6(4-9) |  |  | 47(52.2) | 53(58.2) | 14.5(0,40) | 10(0,50) |  |  |  |  |  |
| Zlatka K 2015 | 7(6-8) | 8(7-8) | M | M |  |  |  |  |  |  |  |  |  |
| Xuewei C 2019 | 9.29±0.82 | 9.29±0.84 | F | F |  |  |  |  |  |  |  |  |  |
| Varaporn S 2014 | <6: 1(3.2%) | 1(3.2%) | M | M | 24(82.7) | 23(74.2) |  |  |  |  |  |  |  |
| FN Sari 2011 |  |  | M | M | 41(36.9) | 31(28.2) | 10 | 11.5 |  |  |  |  |  |
| Ozge S 2013 | 7(6-8) | 8(6-8) | M | M | 73(70.2) | 71(68.3) |  |  | 1.8±1.1 | 2±1 |  |  |  |
| M.N.S 2015 |  |  |  |  |  |  |  |  | 3.12±1.22 | 2.3±0.99 |  |  |  |
| Manish Rasania 2023 |  |  | M | M | 17(42.5) | 25(61.0) |  |  | Day1:18(45.0) | 25(61.0) | 4(10.0) | 1(2.4) |  |
|  |  |  |  | M |  | 20(47.6) |  |  |  | 30(71.4) |  | 1(2.4) |  |

Data are n (%), mean ± SD, median(Q1-Q3), median [IQR], median (min, max), mean [lower-upper 95%CI]. Data are complete except where missing data are detailed.

Abbreviations: FI, feeding intolerance; TPN, total parenteral nutrition. Feeding type*: H, exclusively human milk feeding, not limited to parental or donor milk; F, full formula feeding; M, mixed feeding.

**Table S3: Classification of the included interventions**

| **Interventions** | **Classification** |
| --- | --- |
| Placebo | Placebo |
| Bifidobacterium | B.bifidum  B.breve  B.lactis  B.longum  BB: Bifidobacterium + Bifidobacterium |
| Bacillus | B.clausii |
| Lactobacillus | L.rhamnosus  L.paracasei  L.reuteri  L.sporogenes |
| Saccharomyces | S.boulardii |
| BL | Bifidobacterium + Lactobacillus |
| BLP | Bifidobacterium + Lactobacillus + Prebiotic |
| BSa | Bacillus + Saccharomyces boulardii |
| BSt | Bacillus + Streptococcus |
| BLE | Bifidobacterium + Lactobacillus + Enterococcus |
| BLSa | Bifidobacterium + Lactobacillus + Saccharomyces boulardii |
| BLSt | Bifidobacterium + Lactobacillus + Streptococcus |
| BP | Bifidobacterium + Prebiotic |
| Nystatin | Nystatin |
| Prebiotic | Prebiotic |

**Table S4: Primary outcomes**

|  | **Analyzed [n]** | | **Mortality [n(%)]** | | **NEC [≥Bell's II, n(%)]** | | **Length of hospital stay [day]** | | **Time to reach full feeding [day]** | | **Proven sepsis [n(%)]** | |
| --- | --- | --- | --- | --- | --- | --- | --- | --- | --- | --- | --- | --- |
| **Study** | **Control** | **Intervention** | **Control** | **Intervention** | **Control** | **Intervention** | **Control** | **Intervention** | **Control** | **Intervention** | **Control** | **Intervention** |
| Taciana D 2011 | 112 | 119 | 27(24.1) | 26(21.8) | 4(3.6) | 0(0) |  |  | 17.4±5.7 | 15.2±5.2 |  |  |
| Lingfen X 2016 | 49 | 51 | 0(0) | 0(0) | 0(0) | 0(0) | 28.0±1.8 | 23.3±1.6 |  |  | 6(12.2) | 4(7.8) |
| ALONA2005 | 73 | 72 | 8(11.0) | 3(41.7) | 10(13.7) | 1(13.9) |  |  | 17.5±13.6 | 14.6±8.7 | 28(38.4) | 36(50.0) |
| Dilek D 2015 | 100 | 100 | 12(12.0) | 3(3.0) | 18(18.0) | 2(2.0) | 50(31-70) | 37(27-50) | 25(15-37) | 18(14-23) | 13(13.0) | 8(8.0) |
|  |  | 100 |  | 2(2.0) |  | 12(12.0) |  | 38(27-53) |  | 17(12-24) |  | 10(10.0) |
|  |  | 100 |  | 3(3.0) |  | 4(4.0) |  | 42(33-60) |  | 20(14-30) |  | 8(8.0) |
| Kate C 2016 | 660 | 650 | 56(8.5) | 54(8.3) | 66(10.0) | 61(9.4) | 66(46-95) | 68(48-98) | 14(10-22) | 14(10-22) | 206(31.2) | 186(28.6) |
| Stephane H 2015 | 52 | 50 |  |  | 3(5.8) | 2(4.0) |  |  |  |  |  |  |
|  |  | 48 |  |  |  | 1(2.1) |  |  |  |  |  |  |
|  |  | 47 |  |  |  | 5(10.6) |  |  |  |  |  |  |
| Costalos 2003 | 36 | 51 |  |  |  |  |  |  | 9.9[4.5] | 9.3[2.7] | 3(8.3) | 3(5.9) |
| Manzoni 2006 | 41 | 39 |  |  | 2(4.9) | 1(2.6) | 35±30 | 30±28 | 17±9 | 15±8 | 17(42.5) | 15(37.5) |
| Shashidhar 2017 | 46 | 48 | 3(5.7) | 1(1.9) | 6(12.5) | 2(4.1) | 31.2±22.9 | 27.6±18.5 | 12.7±8.9 | 11.2±8.3 |  |  |
| Elaheh A 2017 | 30 | 30 |  |  | 6(20) | 0(0) |  |  |  |  |  |  |
| Carlo D 2002 | 290 | 295 |  |  | 8(2.8) | 4(1.4) |  |  |  |  | 12(4.1) | 14(4.7) |
| W.A.M 2010 | 89 | 91 | 1(1.1) | 2(2.2) | 4(4.5) | 2(2.2) |  |  | 18.0±7.4 | 17.9±6.8 |  |  |
| M.A.H 2012 | 51 | 50 | 4(7.8) | 3(6.0) | 2(3.9) | 2(4.0) |  |  |  |  | 16(31.4) | 13(26.0) |
| G.A.J 2022 | 29 | 86 | 0(0) | 12(14.0) | 0(0) | 3(3.5) |  |  |  |  | 7(24.1) | 21(24.7) |
|  |  | 87 |  | 8(9.2) |  | 3(3.4) |  |  |  |  |  | 15(17.4) |
| O.S.P 2020 | 104 | 104 | 3(2.9) | 6(5.8) | 4(3.8) | 0(0) | 39(26-56) | 36(26-52) |  |  | 18(17.3) | 27(26.0) |
| Chowdhury 2016 | 60 | 60 |  |  | 6(11.5) | 1(1.9) | 19.57±4.26 | 15.82±2.94 | 18.80±4.32 | 14.88±3.15 |  |  |
| Gamze D 2013 | 136 | 135 | 5(3.7) | 5(3.7) | 7(5.1) | 6(4.4) | 43(4,134) | 47(6,120) | 13.2[11.14-15.40] | 11.7[10.98-12.58] | 21(15.4) | 20(14.8) |
| S Dongol Singh S 2017 | 35 | 37 | 3(8.7) | 3(8.1) | 5(14.3) | 0(0) |  |  |  |  |  |  |
| Fernández 2013 | 75 | 75 | 7(9.3) | 19(1.3) | 12(16.0) | 6(8.0) | 40(8,120) | 45(13,134) | 15(0,39) | 18(0,56) |  |  |
| Moumita S 2009 | 95 | 91 | 14(14.7) | 4(4.4) | 15(15.8) | 5(1.1) | 24.07±4 | 17.17±3.23 | 19.2±2.02 | 13.76±2.28 | 28(29.5) | 13(14.3) |
| E.V.N 2015 | 56 | 54 |  |  | 2(3.6) | 0(0) |  |  | 11.14±4.15 | 9.63±2.42 | 7(12.5) | 10(18.5) |
|  |  | 37 |  |  |  | 2(5.4) |  |  |  | 9.68±3.46 |  | 3(8.1) |
|  |  | 37 |  |  |  | 0(0) |  |  |  | 10.19±4.055 |  | 5(13.5) |
| V.V.T 2015 | 62 | 62 | 5(8.1) | 4(6.5) | 0(0) | 0(0) |  |  |  |  | 8(12.9) | 6(9.7) |
|  |  | 59 |  | 9(15.3) |  | 0(0) |  |  |  |  |  | 17(28.8) |
|  |  | 61 |  | 8(13.1) |  | 0(0) |  |  |  |  |  | 14(23.0) |
| Havranek2013 | 16 | 15 |  |  |  |  |  |  | 22.1±8.5 | 23.9±8.3 |  |  |
| M Strus 2018 | 73 | 80 | 4(5.5) | 2(2.5) | 1(1.4) | 2(2.5) |  |  |  |  | 11(15.1) | 7(8.8) |
| Susan E 2013 | 551 | 548 | 28(5.1) | 27(4.9) | 24(4.4) | 11(2.0) | 74(58-93) | 71(54-92) | 12(10-17) | 12(9-16) | 89(16.2) | 72(13.1) |
| Risma K 2019 | 47 | 47 | 4(8.5) | 1(2.1) | 3(6.4) | 0(0) | 27(11,73) | 27(8,72) | 7(0,63) | 6(0,25) | 3(6.4) | 1(2.1) |
| H.C.L 2005 | 187 | 180 |  |  | 10(5.3) | 2(1.1) |  |  |  |  | 36(19.3) | 22(12.2) |
| H.C.L 2008 | 217 | 217 | 9(4.1) | 2(1.0) | 14(6.5) | 4(1.8) | 43.3±21.0 | 46.4±24.2 | 27.0±18.7 | 29.8±19.7 | 24(11.5) | 40(19.8) |
| Erik W 2018 | 66 | 68 | 5(7.6) | 5(7.4) | 8(12.1) | 7(10.3) |  |  | 15(10-20) | 15(11-23) | 23(34.8) | 25(36.8) |
| Mazyar V 2020 | 53 | 53 |  |  |  |  | 14.30±7.043 | 13.55±6.927 | 10.38±2.719 | 9.70±2.750 |  |  |
| Belal A 2022 | 31 | 31 |  |  |  |  | 96(81-112) | 107.5(91-126) | 13(12-17) | 12(11-19) | 3(9.7) | 8(25.8) |
| L.P.N 2015 | 110 | 108 |  |  | 3(2.7) | 0(0) | 8.40±5.1 | 8.29±4.5 | 2.96±2.92 | 2.17±2.05 | 4(3.6) | 4(3.7) |
| Satsuki T 2014 | 114 | 119 | 0(0) | 2(1.3) | 0(0) | 0(0) | 92.9±40.2 | 92.3±44.5 | 12.1±3.8 | 11.0±3.6 | 10(10.0) | 6(3.9) |
| Flavia I 2017 | 30 | 30 |  |  |  |  | 22.4±3.2 | 13.4±2.2 | 7.5±3.2 | 4.2±1.1 |  |  |
| Marwyn S 2022 | 100 | 100 |  |  |  |  |  |  | 9.7±4.3 | 8.7±2.0 |  |  |
| M.Y.O 2014 | 200 | 200 | 20(10.0) | 15(7.5) | 10(5.0) | 8(4.0) | 46(10,180) | 38(10,131) | 10.1±4.3 | 9.1±3.2 | 25(12.5) | 13(6.5) |
| M.Y.O 2015 | 150 | 150 | 16(10.7) | 12(8) | 9(6.0) | 7(4.7) | 48.4±29.2 | 42.4±24.1 | 10.4±4.7 | 9±3.1 | 22(14.7) | 11(7.3) |
| İpek Güney V 2017 | 40 | 70 | 9(22.5) | 1(1.4) | 4(10.0) | 0(0) |  |  | 21±8 | 14±3 | 14(35.0) | 12(17.1) |
| M.A.R 2012 | 378 | 372 | 28(7.4) | 22(5.9) | 15(4.0) | 9(2.4) | 20.0(11-38) | 20.0(11-33) |  |  | 40(10.6) | 34(9.1) |
| Sanjay P 2014 | 76 | 77 | 0(0) | 0(0) | 1(1.3) | 0(0) | 10(7-14) | 10(6-14) | 12(8-16) | 12(9-21) | 12(15.8) | 17(22.1) |
| SourabhD 2015 | 35 | 38 | 2(5.7) | 3(7.9) | 0(0) | 1(2.6) |  |  |  |  | 6(17.1) | 3(7.9) |
|  |  | 38 |  | 3(7.9) |  | 3(7.9) |  |  |  |  |  | 1(2.6) |
|  |  | 38 |  | 2(5.3) |  | 2(5.3) |  |  |  |  |  | 6(15.8) |
| Ghasem B 2021 | 38 | 38 |  |  | 7(18.4) | 0(0) | 10.37±4.58 | 7.87±4.53 |  |  |  |  |
| Mahtab M 2022 | 26 | 26 | 0(0) | 0(0) |  |  | 27.6±11.8 | 23.6±11.5 | 31.9±15.7 | 22.5±12.3 | 3(11.5) | 0(0) |
|  |  | 26 |  | 0(0) |  |  |  |  |  |  |  |  |
| Gamze D 2013 | 90 | 91 | 2(2.2) | 2(2.2) | 2(2.2) | 2(2.2) | 50.5(8,134) | 50.5(10,120) |  |  |  |  |
| Zlatka K 2015 | 40 | 40 | 3(7.5) | 2(5.0) | 5(12.5) | 0(0) | 67.0(54.5-95.0) | 61.0(46.7-76.2) | 31.0(22.2-41.5) | 28.0(21.5-40.5) | 29(72.5) | 16(40.0) |
| Xuewei C 2019 | 48 | 45 |  |  |  |  | 23.75±8.57 | 20.60±5.36 | 13.80±3.47 | 9.95±2.46 | 4(8.3) | 2(4.4) |
| Varaporn S 2014 | 29 | 31 | 0(0) | 0(0) | 1(3.4) | 1(3.2) | 56.9±27.19 | 59.84±31.96 | 13.76±8.25 | 12.03±5.49 | 1(3.4) | 2(6.5) |
| FN Sari 2011 | 111 | 110 | 4(3.6) | 3(2.7) | 10(9.0) | 6(5.5) | 30 | 34.5 | 18.3±9.8 | 17.3±8.7 | 26(23.4) | 29(26.4) |
| Ozge S 2013 | 104 | 104 | 4(3.8) | 5(4.8) | 7(6.7) | 7(6.7) | 43(29-60) | 39(28-60) | 12±7 | 11±7 | 25(24.3) | 19(18.3) |
| M.N.S 2015 | 30 | 30 | 2(6.7) | 1(3.3) |  |  |  |  | 16.75±6.592 | 12.83±4.268 | 10(33.3) | 4(13.3) |
| Manish Rasania 2023 | 40 | 41 | 1(2.5) | 0(0.0) | 0(0) | 1(2.4) | 20.38±14.6 | 14.11±10.95 | 5.08±2.07 | 4.61±1.56 | 3(7.5) | 5(12.2) |
|  |  | 42 |  | 1(2.4) |  | 1(2.4) |  | 16.1±10.67 |  | 4.19±1.74 |  | 3(7.1) |

Data are presented as n (%), mean ± SD, median(Q1-Q3), median [IQR], median (min, max), mean [lower-upper 95%CI]. Data are complete except where missing data are detailed.

Abbreviations: NEC, necrotizing enterocolitis.
